# Genetic Variants in *REC8*, *RNF212*, and *PRDM9* Influence Male Recombination in Cattle

**DOI:** 10.1371/journal.pgen.1002854

**Published:** 2012-07-26

**Authors:** Cynthia Sandor, Wanbo Li, Wouter Coppieters, Tom Druet, Carole Charlier, Michel Georges

**Affiliations:** Unit of Animal Genomics, GIGA-R and Faculty of Veterinary Medicine, University of Liège, Liège, Belgium; The Jackson Laboratory, United States of America

## Abstract

We use >250,000 cross-over events identified in >10,000 bovine sperm cells to perform an extensive characterization of meiotic recombination in male cattle. We map Quantitative Trait Loci (QTL) influencing genome-wide recombination rate, genome-wide hotspot usage, and locus-specific recombination rate. We fine-map three QTL and present strong evidence that genetic variants in *REC8* and *RNF212* influence genome-wide recombination rate, while genetic variants in *PRDM9* influence genome-wide hotspot usage.

## Author Summary

Homologous recombination is an essential cellular process that determines proper chromosome segregation during meiosis, affects fertility, and influences evolvability. Nevertheless, the components of the recombination apparatus remain incompletely characterized in mammals. One approach to identify such components is to identify the genes that underlie inherited variation in recombination phenotypes. In addition to providing mechanistic insights, this would allow the study of the evolutionary forces that shape the recombination process. In this paper, we take advantage of genotypes for 50,000 genome-wide SNP markers to measure four recombination phenotypes (genome-wide recombination rate, genome-wide hotspot usage, locus-specific recombination rate, genome-wide cross-over interference) for >750 bulls on the basis of >250,000 cross-overs detected in sperm cells transmitted to >10,000 sons. We quantify the heritability and scan the genome for Quantitative Trait Loci (QTL) influencing each one of these recombination phenotypes. We perform a detailed genetic analysis of three such QTL, thereby providing evidence that genetic variants in *REC8* and *RNF212* influence genome-wide recombination rate, while genetic variants in an X-linked *PRDM9* paralogue influence genome-wide hotspot usage.

## Introduction

Reciprocal recombination between homologues fulfills an essential mechanistic role during meiosis in most organisms [1], [2]. It is required for proper bivalent alignment on the metaphase I plate preceding disjunction and segregation at anaphase I. Correct segregation of the full chromosome complement demands tight, sex-specific control of the number of cross-overs (CO) per arm, as well as of their position relative to chromosomal landmarks (centromeres and telomeres) and other CO (in the case of multichiasmatic meioses) [3], [4]. Failures in this process underlie aneuploidies affecting as many as 5% of human oocytes [5].

At the population level, recombination affects the rate of creation and loss of haplotypes with *cis*-configured favorable alleles, placing second order selection pressure on modifiers of global and/or local recombination including inversions [3].

Components of the recombination apparatus are well described in yeast and *C. elegans*, but remain largely undefined in most other organisms including mammals [4], [6]. One strategy to identify such components is to positionally clone the genes and variants that underlie inherited variation in recombination phenotypes. Genome-wide recombination rate (GRR) is characterized by considerable inter-individual variation, which is in part inherited [7]–[10]. Genome-wide association studies (GWAS) have identified several loci influencing GRR in human [11]–[13]. These include the 17q21.31 inversion [11], as well as the *RNF212* gene harboring common variants with antagonistic effects on GRR in males and females [12]. Of note, women's recombination rate correlates positively with reproductive success^9^. In human, ∼80% of CO events map to ∼10–20% of the genome, encompassing >25,000 recombination hotspots [3], [14]–[16]. Hotspot usage differs considerably between individuals [17] and this was shown to involve variation in *cis*-acting hotspot-triggering sequences [18], as well as in the *trans*-acting *PRDM9* H3K4 trimethyltransferase and hotspot regulator [19]–[22]. Recombination hotspots and their *PRDM9* regulator undergo accelerated evolution (explained in part by the self-destructive drive of hotspot motifs due to biased gene conversion) [18], [21], [23], [24], and *PRDM9* has been identified as a hybrid sterility gene in the mouse [25]. Genome-wide levels of cross-over interference were also suggested to differ between individuals [26], [27], but corresponding genetic variants – if existing - have not been identified thus far.

We herein describe our efforts to take advantage of (i) the large multigenerational half-sib pedigrees typifying dairy cattle population and (ii) the systematization of genome-wide SNP genotyping with ∼50 K medium density arrays for “genomic selection" purposes [28], to quantify inter-individual variation in recombination phenotypes as well as to map contributing genetic loci. The bovine haploid genome is estimated at 2.87 Gbp distributed over 29 acrocentric chromosomes and a pair of metacentric sex chromosomes [29]. Total map length was previously estimated at ∼31M and shown (contrary to most other mammals) not to differ between sexes [30]. The potential correlation between recombination rate and fertility, as well as the hypothesized effect of domestication on recombination rates [31] adds to the interest of a detailed characterization of recombination phenotypes in livestock.

## Results

### Characterizing recombination in male cattle

**Figure 1:**
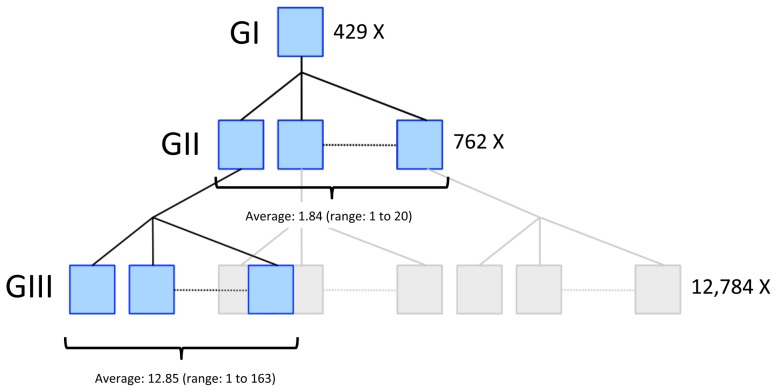
Three-generational pedigrees used to map genetic determinants of variation in male recombination rate in cattle. 10,192 (Dutch population) and 3,783 (NZ population) bulls, genotyped for 50K SNP panels, assorted in 429 three-generational pedigrees of the kind illustrated. All Dutch bulls were from the Holstein-Friesian breed, while in NZ 61% of the bulls were Holstein-Friesian and 39% Jerseys. Each pedigree is composed of one grand-sire with 1.84 GII sons on average (range: 1 to 20). Each GII sire has 12.85 GIII sons on average (range: 1 to 163). We have used the available SNP genotypes to identify 259,752 CO events that occurred in the sperm cells transmitted by the GII sires to their 10,106 GIII sons. QTL affecting variation in recombination rates were mapped by exploiting linkage information (effect of the homologues transmitted by the GI grand-sires to their GII sons) and LD information (effect of haplotypes transmitted by the GI-grand-sires and ungenotyped GI-grand-dams to their GII sons).

The dataset available for analysis comprised 10,192 bulls from the Netherlands (H) and 3,783 bulls from New-Zealand (NZ), that were genotyped for marker panels comprising respectively 50,876 [32] and 51,456 [33] SNPs of which 19,487 in common. The 13,975 bulls assorted in 429 three-generational paternal half-sib pedigrees of the structure shown in Figure 1. All Dutch bulls were from the Holstein-Friesian (HF) breed, while in NZ 61% of the bulls were HF and 39% Jerseys (J). SNP genotypes were phased [34], and CO events identified in the gametes transmitted by generation II (GII) bulls to their GIII sons. We identified 259,752 CO in 10,106 gametes, corresponding to an average genome size of 25.7 M(organs).

Average number of CO for each of the 29 acrocentric chromosomes was remarkably well predicted (r^2^ = 0.96) by (i) size in bp (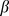
_1_ = 0.07CO/10 Mb) and (ii) the requirement for at least one chiasma per meiosis (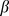
_0_ = 0.48 CO) (Figure S1A). Also in agreement with the obligate chiasma theory, the frequency distribution of gametes with 0, 1, 2, … CO-events was best explained [35] assuming near absence of nullichiasmatic meioses for all autosomes. Moreover, under a truncated Poisson model forcing the proportion of nullichiasmatic meioses at zero [36], the most likely frequency of meioses with one chiasma was considerably lower than expected, and this was largely due to an excess of meioses with two chiasmata. This supports the preferred occurrence of a second chiasma, particularly for the larger chromosomes (Figure S1B).

Recombination rate (RR) computed in 60-Kb windows averaged 0.00062 (i.e. ∼1 cM/1 Mb), but was strongly over-dispersed with an excess of “hot" and “cold" windows (defined as windows with RR>2.5 standard deviations from the mean) (Figure S2A–S2C). Note that hot windows as defined here (60 Kb) cannot be compared with recombination hotspots as defined in human and mouse genetics (≤5 Kb) [14], [15], [37]. On average, 34% of CO events could be assigned to hot windows representing 13% of the genome. Hot and cold windows differed in base pair composition and repeat content (Table S1). Hot windows tended to concentrate in sub-terminal (proximal chromosome end) and terminal regions (distal chromosome end), while cold windows concentrated in the middle of the chromosome arms as well as in terminal regions (proximal chromosome end) coinciding with the centromeres (Figure S2D). Hot and cold windows tended to cluster in what we refer to (following Chowdhury et al. [13]) as recombination “jungles" and “deserts", respectively.

We measured chromosome-specific levels of cross-over or chiasma interference using the shape parameter (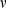) of a gamma distribution [26], [38]. We used a maximum likelihood approach extracting information from the frequency distribution of (i) the number of CO per gamete, (ii) CO-position (in centimorgan (cM)) for gametes with one CO, (iii) inter-CO distance (in cM) for gametes with two CO, and (iii) inter-CO distance (in cM) for gametes with three CO. Positive interference was evident for all chromosomes, manifesting itself by (i) a paucity of gametes with zero CO, (ii) less uniform than expected distribution of single CO position, and (iii) inflated distance between CO for gametes with multiple CO. The value of 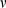 that maximized the overall likelihood averaged 2.6 (range: 1.5–3.1) across all chromosomes (versus 4.5 in human [26]). It was primarily determined by the inter-CO distance for gametes with two recombination events. Values of 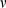 maximizing the likelihood of the frequency distribution of number of CO events and of CO-position for gametes with one recombination tended to be larger that the value of 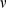 maximizing the likelihood of the inter-CO distance for gametes with two recombinations, while values of 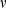 maximizing the likelihood of the distance between CO for gametes with three recombinations tended to be smaller. Of note, the observed distribution of CO events per gamete and hence of chiasmata per meiosis, was remarkably well accounted for by positive interference. There was no evidence for an effect of chromosome length on 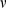, whether maximizing the overall likelihood or that of the constituent parameters (Figure S3A–S3B).

### Genetic analysis of genome-wide recombination rate (GRR)

Average genome-wide recombination rate (GRR) (corrected for family size - M&M) differed significantly between GII sires (p<0.0001; range: 18.7–32.1)(Figure S4A). We took advantage of the fact that 72 of the GII bulls had non-overlapping sets of GIII sons in H and NZ, to estimate the repeatability of GRR as the correlation between these independent measurements, yielding a highly significant Spearman's correlation coefficient of 0.58 (p<3.7×10^−7^)(Figure S4B). We estimated the heritability (*h^2^*) of GRR at 0.22 in the Dutch HF breed.

**Figure 2:**
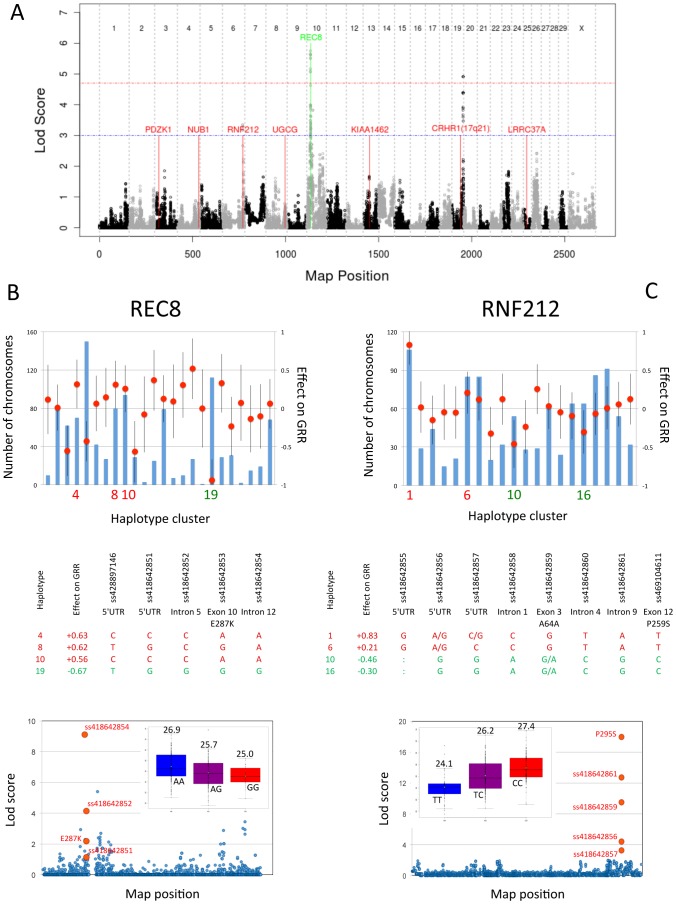
Genome-wide lod score profiles, observed counts and effects on global recombination rate. (A) Genome-wide lod score profiles obtained for GRR in the Holstein-Friesian (H+NZ) sample set. The red and blue horizontal lines mark the genome-wide significant and suggestive thresholds determined by permutation testing. The position of seven loci that have been previously implicated as determinants of variation in GRR [12], [13] are shown in red, while the position of *REC8* is shown in green. (B) Observed counts (blue bars), and effects on global recombination rate (GRR) (red circles) ± standard error (black vertical lines) for 25 hidden haplotype clusters at map position of *REC8* gene. Effect on GRR and genotype at five DNA sequence variants in *REC8* for four sequenced hidden haplotype clusters. Combined linkage+LD analysis of BTA10 SNPs on GRR. Sequence variants in *REC8* are shown in red. The inset shows the distribution of GRR for GII sires sorted by ss418642854 genotype, with indication of the average GRR (in M) per genotype. (C) Observed counts (blue bars), and effects on global recombination rate (GRR) (red circles) ± standard error (black vertical line) for 20 hidden haplotype clusters at map position of *RNF212* gene. Effect on GRR and genotype at five variant positions in *RNF212* for four sequenced hidden haplotype clusters. Combined linkage+LD analysis of BTA6 SNPs on GRR. Sequence variants in *RNF212* are shown in red. The inset shows the distribution of GRR for GII sires sorted by ss469104611 ( = P259S) genotype, with indication of the average GRR (in M) per genotype.

We used a Hidden Markov Model-based approach that simultaneously exploits linkage and linkage disequilibrium [34] to scan the genome for QTL influencing GRR. At each SNP position, all chromosomes in the dataset (i.e. 2*n* chromosomes for a data set with *n* animals) were assigned to one of 20 hidden states corresponding to “ancestral haplotype states". The effect of these hidden haplotype states (HHS) on the GRR was then estimated using a mixed model including a polygenic effect to correct for population stratification (M&M). We only used HF animals (from both H and NZ) in these analyses. We identified two genome-wide significant QTL, respectively on BTA10 (z = 5.8) and BTA19 (z = 4.9)(Figure 2A).

The lod-2 drop-off confidence interval (CI) of the BTA10 QTL spanned ∼1.4 Mb encompassing 47 genes. Three of these are strongly expressed in testis: *TBC1D21*, *TSSK4*, and *REC8*. *REC8* is a particularly appealing positional candidate as it codes for a member of the kleisin family of SMC (structural maintenance of chromosome) proteins, which localizes to the axial elements of chromosomes during meiosis in both oocytes and spermatocytes. The mouse homologue is a key component of the meiotic cohesion complex, which regulates sister chromatid cohesion and recombination between homologous chromosomes [39], [40]. We therefore re-sequenced 7.2 Kb encompassing the *REC8* gene (including 1.2 Kb upstream of the start codon and 0.9 Kb downstream of the polyadenylation site; Figure S5A and Table S2) for animals selected to obtain the sequence of three HHS associated with an increase in GRR and one associated with a decrease in GRR (as HHS with divergent effect on GRR should differ at the causative variant positions)(Figure 2B). We identified five SNPs located respectively in the 5′UTR (ss428897146 and ss418642851), intron 5 (ss418642852), exon 10 (ss418642853 = E287K) and intron 12 (ss418642854). Of note, two of these (ss418642852 and ss418642854) segregated perfectly between the high and low GRR haplotypes. We developed 5′exonuclease assays for ss418642851, ss418642852, ss418642853 and ss418642854 (Table S3), and genotyped the GI and GII sires. We performed single point association analysis using a mixed model including the (random) effect of the SNPs as well as a polygenic animal effect to correct for stratification. Ss418642854 yielded a lod score of 9.12, i.e. 3.7 units higher than any other BTA10 SNP, and 3.3 units higher than the highest BTA10 haplotype-based signal. The difference in GRR between alternate homozygotes at the ss418642854 SNP was 1.8 CO/genome (Figure 2B). To provide additional support for the causality of the *REC8* gene, we took advantage of the fact that 121 HF GII sires had also been genotyped with a recently developed high-density Illumina 777K SNP array, including 45 SNPs spanning the QTL CI. When performing single point association analysis using the same mixed model for all SNPs in the CI, the lod score still clearly maximized on top of the *REC8* gene and for SNP ss418642854 (Figure S4D). Taken together, these results support the fact that variation in the *REC8* gene indeed underlies the identified QTL.

The CI of the BTA19 QTL spans ∼0.6 Mb encompassing two genes: *KCNJ2* and *KCNJ16*. Neither is knowingly related to recombination, yet both are expressed in testes (data not shown). Preliminary sequence analysis of the *KCNJ2* and *KCNJ16* open reading frames (ORF) of animals carrying haplotypes with significantly different effect on GRR did not reveal obvious variants that might underlie the observed effects (data not shown).

In addition to these two significant QTL, we obtained a suggestive lod score of 3.2 on BTA6 that maximized at the exact position of the *RNF212* gene (Figure 2A). This suggests that variation in *RNF212* affects GRR in cattle as it does in human [12], [13]. Homologues of *RNF212* in *C. elegans* (*ZHP3*) and yeast (*ZIP3*) are known to be involved in meiotic recombination [12]. We re-sequenced 10 amplicons encompassing the entire *RNF212* ORF and intron-exon boundaries (Figure S5B and Table S2) in animals selected to obtain the sequence of two haplotypes increasing and two decreasing GRR (Figure 2C). We identified eight SNPs located respectively in the 5′UTR (ss418642855, ss418642856 and ss418642857), intron 1 (ss418642858), exon 3 (ss418642859), intron 4 (ss418642860), intron 9 (ss418642861) and exon 12 (ss469104611 = P259S). Five of these (ss418642855, ss418642858, ss418642860, ss418642861 and P259S) segregated perfectly between the high and low GRR haplotypes. We developed five 5′exonuclease assays (Table S3) and genotyped the GI and GII sires. Ss469104611 ( = P259S) yielded a lod score of 18, i.e. 15.3 units higher than any other BTA6 SNP and 14.8 units higher than the highest BTA6 haplotype-based signal. The difference in GRR between alternate homozygotes at the P259S variant was 3.3 CO/genome (Figure 2C). We took advantage of the same 121 GII sires genotyped with the high-density 777K Illumina array, including 27 SNPs in the ∼1 Mb CI of the BTA6 QTL. Lod scores clearly maximized on top of the *RNF212* gene, at the position of the ss469104611 variant (Figure S4E). Taken together, these results strongly supported the causality of the *RNF212* gene.

### Genetic analysis of genome-wide hot window usage (GHU)

We then computed, for each GII bull, the proportion of CO falling in hot windows (i.e. the genome-wide hot-window usage or GHU). GHU differed significantly between GII sires (p<0.002; range: 4%–58%), was repeatable (Spearman's correlation: 0.46; p<0.0008) and had a heritability of 0.21 in Dutch HF (Figure S6).

**Figure 3:**
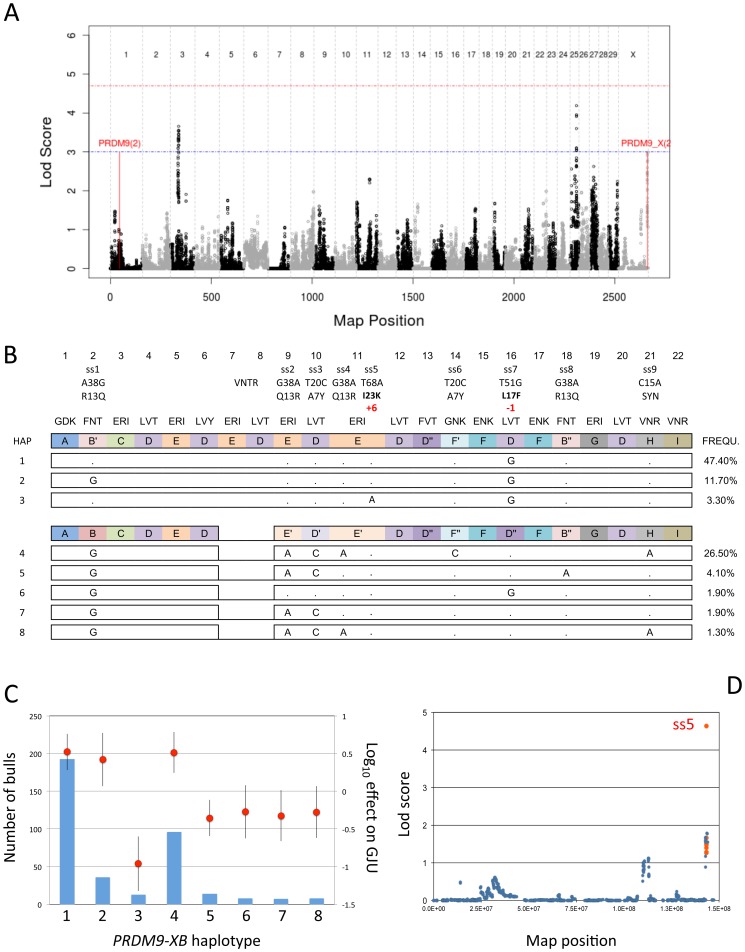
Results of the genome-scan, polymorphisms, observed counts and effects, results of single point analysis. (A) Results of genome-scan for QTL affecting genome-wide jungle usage (GJU) using a method that simultaneously extracts linkage and LD signal [34]. The red and blue horizontal lines mark the genome-wide significant and suggestive thresholds determined by permutation testing. The position of two pairs of PRDM9 parologues, respectively on BTA1 and BTAX, are highlighted. (B) Polymorphisms detected in the ZF array of the *PRDM9-XB* paralogue. Nine SNP are labeled ss1 to ss9, while the length polymorphism corresponding to the loss of two ZF domains is labeled VNTR. For each SNP, we define the position and the nature of the nucleotide substitution in the corresponding ZF domain (labeled in top row). For non-synonymous substitutions, we also define position and nature of the amino-acid substitution. Ss9 is synonymous and labeled as such (SYN). We represent the eight detected haplotypes sorted by VNTR genotype: haplotypes with 22 ZFs above, haplotypes with 20 ZFs below. For each length class, the top row represents the corresponding ZF domains labeled A, B, C, … I, and colored accordingly. ZFs differing by ‘or ‘’ (f.i. D, D′ and D″) have an amino-acid similarity of ≥92%. The triplet of amino-acids at DNA binding positions −1,3 and 6 ^f.i.41^ are shown above each ZF. The frequency of the eight haplotypes in the HF population are given in the column on the right. (C) Observed counts (blue bars), and effects on genome-wide jungle usage (log_10_ of GJU) (red circles) ± standard error (black vertical line) for the eight *PRDM9-XB* haplotypes defined in (B). (D) Result of single point analysis on GJU for BTAX. The ten *PRDM9-XB* variants are highlighted in red.

We scanned the genome for QTL affecting GHU in HF, and identified three suggestive QTL, respectively on BTA3 (z = 3.7), BTA25 (z = 4.1) and BTAX (z = 2.8)(Figure 3A). The CI of the BTA3 QTL spans ∼2.1 Mb and encompasses three genes (*LOC781798*, *LOC522984* and *OLFM3*) not obviously related to recombination. The CI for the BTA25 QTL (UMD3 31.29–33.62 Mb) contains 25 genes of unknown function.

Most interestingly, the lod score peak on the X chromosome coincided with the position of two adjacent gonosomal *PRDM9* paralogues (hereafter referred to as *PRDM9-XA* and *–XB*). In mice and human, genome-wide hotspot usage has been shown to be genetically controlled, with variation in the *PRDM9* C-terminal tandem array of Cys_2_His_2_ zinc-finger (ZF) domains having a major effect [19]–[22]. We therefore designed amplicons allowing specific amplification and sequencing of the complete *PRDM9-XA* and *-XB* ZF arrays. The C-terminal ZF arrays of the *PRDM9-XA* and *PRDM9-XB* reference sequences (UMD3 build) contain respectively eight and 20 ZF domains in tandem (Table S2). Sequence analyses indicate that bovine *PRDM9* ZF arrays are rapidly evolving (as they are in human and rodents but not in dogs [41]–[43]), and this is predicted to increase allelic heterogeneity. We thus decided to determine the sequence of the *PRDM9-XA* and *–XB* ZF arrays for 80 individuals representing all 20 hidden haplotype states. Not a single polymorphism, whether synonymous or not, was observed for the *PRDM9-XA* array. For *PRDM9-XB*, however, we detected (i) a VNTR-like length polymorphism (as we detected a common allele with 22 ZF), and (ii) nine SNPs (Figure 3B). Notably, eight of the nine SNPs were non-synonymous. Two affected residues that are predicted to mediate DNA binding, located respectively in ZF 11 out of 22 (11/20) (ss5 = I23K, position +6) and 16/22 (ss7 = L17T; position -1). Four corresponded to R<->Q amino-acid substitutions at position 13 of ZF 2/22 (ss1), 9/22 (ss2), 11/22 (ss5) and 18/22 (ss8). Two corresponded to A<->Y amino-acid substitutions at position 7 of ZF domains 10/22 (ss3) and 14/22 (ss6). Based on these results, we decided to sequence the *PRDM9-XB* ZF array for all GI and GII sires. The 10 polymorphisms assorted in eight haplotypes observed at least five times, jointly accounting for 98.6% of the sequenced chromosomes (Figure 3B). We tested the effect of *PRDM9-XB* haplotype on GHU using the mixed model described above, and obtained a lod score of 7.3, i.e. 4.5 units higher than in the initial scan, hence strongly supporting the causality of the *PRDM9-XB* paralogue. Analysis of the effects of individual haplotypes indicates that: (i) ss5 has a major effect, the K allele decreasing GHU ∼30-fold when compared to the I allele (hap1-hap3 contrast), (ii) ss1 has no effect on GHU (hap1-hap2 contrast), (iii) the VNTR affects GHU as the loss of two ZFs decreases GHU ∼6-fold (hap2-hap6 contrast), (iv) ss2, ss3, ss4, ss7, ss8 and ss9 have no effect on GHU (hap6-(hap5,hap7,hap8) contrasts), (v) ss6 affects GHU, the Y allele increasing GHU ∼6-fold when compared to the A allele (hap4-hap8 contrast)(Figure 3C). The major effect of the ss5 variant was also apparent from single-point analyses, yielding a lod score of 4.6 (Figure 3D).

### Genetic analysis of locus-specific recombination rate (LRR)

**Figure 4:**
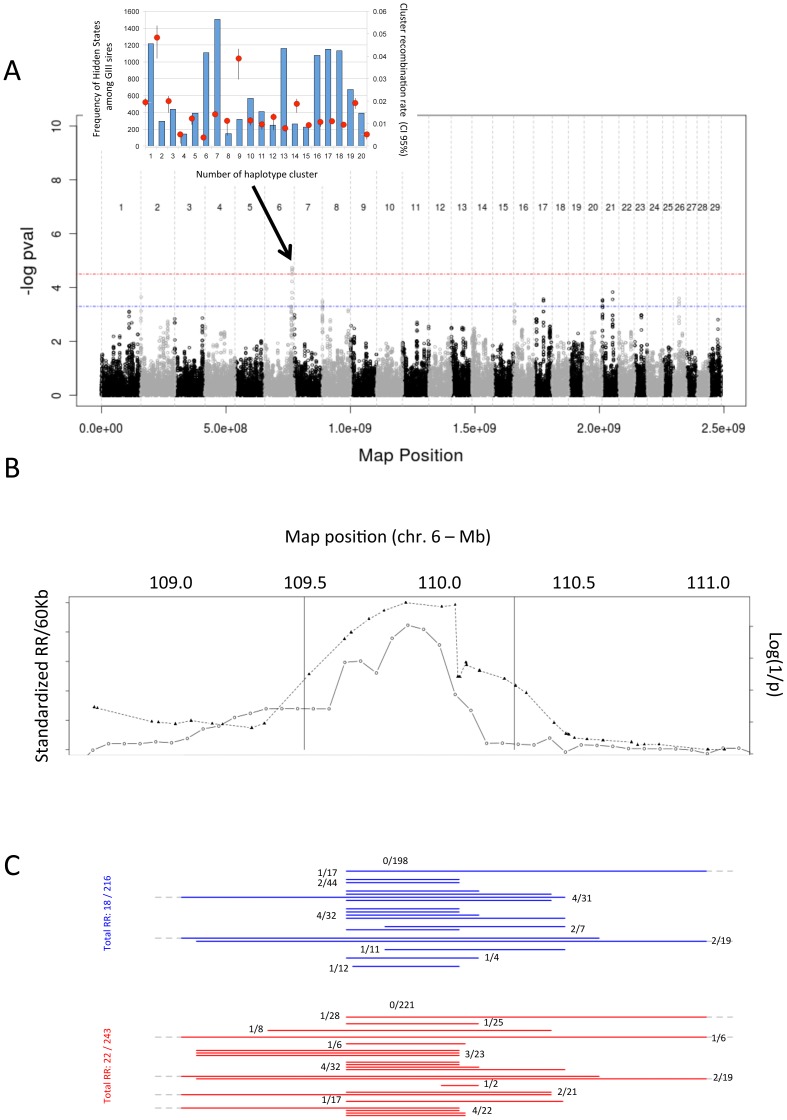
Log(1/p) values for *cis*-acting haplotype effects on local recombination rate, normalized recombination rate, and marker intervals. (A) Log(1/p) values for *cis*-acting haplotype effects on local recombination rate. The horizontal lines correspond to the significant (red) and suggestive (blue) thresholds. The inset shows the frequency (blue bars, left axis) and effect on recombination rate (red circles, right axis) with 95% CI obtained by bootstrapping (black vertical lines) for the 20 modeled hidden haplotype clusters at the most significant BTA6 position. (B) Normalized recombination rate (triangles, dotted line) and log(1/p) (circles, continuous line) values around the BTA6 QTL. The vertical lines mark the limits of the 800 Kb window in which the *cis*-acting haplotype effect was strongest. (C) Marker intervals to which the recombination events underlying the QTL were mapped. Recombinant individuals are sorted by GII sire (red: heterozygous for HS 2; blue: heterozygous for HS 9) with indication of the number of recombinant/total number of GIII sons for the corresponding GII sire. Numbers were summed for all GII sires without GIII sons recombining in the interval of interest (0/198 and 0/221).

Rapid *PRDM9* evolution presupposes accelerated turn-over and hence high polymorphism of recombination hotspots [41]. To test this hypothesis, we scanned the genome for *cis*-acting haplotype effects on LRR (in HF). We tested the effect of hidden haplotype state of the GII sires on the recombination rate in an 800-Kb window centered on the interrogated SNP position (M&M). We obtained one genome-wide significant effect on BTA6 (Figure 4A). The observed signal was primarily driven by two haplotype clusters (HS2 and HS9), increasing recombination ∼4 to 5-fold (Figure 4A). The association signal maximized in the middle of a 840-Kb recombination jungle, for which the observed recombination rate exceeded expectation by up to ∼8.5 SD (Figure 4B). LRR in the corresponding 800-Kb window was of the order of 8–9% for GII sires heterozygous for either the HS2 or HS9 haplotypes.

Eight additional peaks exceeded the genome-wide suggestive threshold (by definition, expected by chance only once per genome scan), supporting the common occurrence of *cis*-acting haplotype effects on local recombination rate, and presumably reflecting polymorphisms in *cis*-acting recombination-triggering motifs [18].

### Genetic analysis of genome-wide interference (GIL)

**Figure 5:**
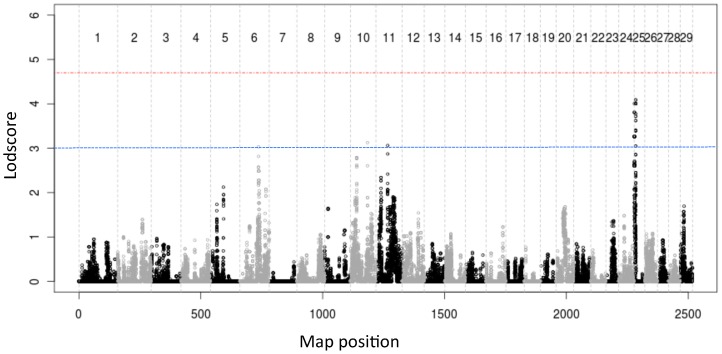
Results of genome-scan for QTL affecting the normalized distance between pairs of CO events measured in base-pairs (GIL_bp_), using a method that simultaneously extracts linkage and LD signal [**34**]. The red and blue horizontal lines mark the genome-wide significant and suggestive thresholds determined by permutation testing.

We finally evaluated inter-individual variation in genome-wide interference levels (GIL). As interference levels were primarily determined by inter-CO distance for gametes with two CO (cfr. above), we used this metric for QTL mapping. Distances between CO were measured both in centimorgan (GIL_cM_) and base-pairs (GIL_bp_), and expressed in standardized deviations from the chromosome mean. Both measures proved to significantly differ between GII sires (GIL_cM_: p<0.002; GIL_bp_: p<0.001), to be repeatable (GIL_cM_: 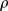 = 0.36, p<0.03; GIL_bp_: 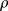 = 0.53, p<0.00003) but to have low heritability (GIL_cM_: 0.045; GIL_bp_: 0.052) in Dutch HF (Figure S7). We nevertheless scanned the genome for QTL affecting GIL in HF. We identified no QTL when using GIL_cM_, yet one genome-wide suggestive QTL (z = 4.1) on BTA25 when analyzing GIL_bp_. The CI of the QTL encompassed four genes (*E-NPP7*, *LOC100297064*, *FOX1* and *TMEM114*) not knowingly involved in recombination (Figure 5; Figure S7).

## Discussion

We herein estimate the male map length in domestic cattle at 25.7 Morgan based on the analysis of CO events in >10,000 sperm cells. This is ∼5 M lower than previous estimates [30], but in better agreement with the relationship between number of chromosome arms and map length observed in other species [3]. Our findings suggest re-evaluation of (i) the presumed equal male and female recombination rate in cattle, and (ii) the inflation of recombination as a result of domestication.

We demonstrate that GRR is repeatable, that it differs between sires, and that ∼21% of the observed variation is inherited in the HF breed. We identify two significant and one suggestive QTL influencing GRR. We provide evidence that two strong positional candidate genes, namely *REC8* and *RNF212*, are very likely causative. We reach this conclusion by targeting resequencing efforts to haplotype clusters with significantly different effect on GRR, leading to the identification of SNPs that exhibited highly significant increases in association signal. While variation in *RNF212* has been previously shown to affect GRR in human [12], [13], the implication of *REC8* is novel. For *RNF212*, the variant yielding the strongest association is a missense variant resulting in a proline to serine substitution. Despite the fact that the corresponding protein segment is poorly conserved, P259S is a strong candidate causative variant. However, we cannot exclude that the causative variant is regulatory, lying outside of the sequenced *RNF212* segments and in LD with P259S, nor that additional causative *RNF212* variants exist. For *REC8*, the causative variants are most likely regulatory, as coding variants strongly associated with GRR could not be detected despite the sequencing of haplotypes with opposite effects. The most strongly associated SNP (ss418642854) is potentially causal, although the affected sequence is not strongly conserved. Thus, it remains possible that other variants outside the sequenced regions will show equal or even stronger association with GRR. Further sequencing and functional studies are required to achieve complete molecular understanding of these two QTL.

Confirming previous findings in human and mice, we observed an overdispersion of LRR, CO tending to preferentially occur in hot windows (exhibiting sequence features reminiscent of human recombination hotspots), while avoiding cold windows. As expected from human and mouse, hot windows tended to concentrate in sub-terminal regions, while cold windows were enriched at centromeres and in the middle of chromosome arms. The propensity for CO to occur in hot windows (GHU) was shown to be a repeatable and heritable phenotype in HF (h^2^≈21%). We identified three genomic loci with suggestive evidence for an effect on GHU. Strikingly, one of these co-localized with two X-linked *PRDM9* paralogues. By resequencing bulls representing all hidden haplotype clusters, we identified nine SNPs and a VNTR-type polymorphism in the *PRDM9-XB* paralogue. Using a haplotype-based approach, we provide strong evidence that an I to K substitution at DNA binding position +6 of ZF 11 decreases GHU ∼30-fold, without affecting GRR. Moreover, we provide suggestive evidence that the VNTR-like polymorphism as well as an A to Y amino-acid substitution at position 7 of ZF domain 14 independently modulate GHU ∼6-fold. Surprisingly, four of the eight non-synonymous variants correspond to R<->Q substitutions at amino-acid position 13 of four distinct ZF domains. None of these variants appear to affect GHU. While this could indicate that the corresponding position is highly mutagenic, we believe that it is more likely that this finding reflects the spreading of a variant within the ZF array by a process of concerted evolution of tandem repeats [44]. Likewise, ss3 and ss6 both correspond to A<->Y substitutions at amino-acid position 7. Surprisingly, no polymorphisms were observed in the equivalent (although shorter) *PRDM9-XA* array. The reason for this striking difference remains unknown, especially given the fact that both *PRDM9-XA* and *PRDM9-XB* appear to be expressed in bovine testes (data not shown).

In further support of the rapid coevolution of *PRDM9* and recombination hotspots in the bovine, we identify haplotypes with significantly different propensity to engage in recombination at a specific BTA6 jungle. We hypothesize that this results from sequence differences at recombination triggering motifs. This model predicts epistatic interactions between *PRDM9* variation and BTA6 haplotype, and analyses to uncover such effects are ongoing.

We demonstrate that, as expected, all chromosomes are subject to positive interference, multiple CO being more distant that expected by chance alone. By applying a gamma-model to the distance between MLH1 foci, Lian et al. [27] observed that interference might increase with decreasing chromosome size. It was subsequently indicated, however, that the observed trend might be due to inappropriate modeling of finite chromosome size [45]. It has been suggested that crossovers might involve two pathways [f.i. 46]: (i) the pairing pathway not subject to interference, and (ii) the disjunction pathway undergoing interference. As the proportion of pairing over disjunction CO increases with decreasing chromosome size, the two-pathway model predicts a decrease in interference levels with decreasing chromosome size as observed in budding yeast [47]. However, we did not find evidence for an effect of chromosome size on levels of interference, in general agreement with Broman and Weber [26] for human. We devised a novel metric to quantify genome-wide interference, and showed that it is repeatable and differs significantly between individuals, yet modestly heritable. We obtain preliminary evidence for the existence of a QTL influencing this trait on BTA25. The corresponding signal was observed when measuring inter-CO distance in base pairs but not when measured in centimorgan. Further studies will be required to verify the genuine nature of this QTL.

## Methods

### Identifying CO events and data cleanup

Marker phasing was conducted with the Phasebook software package [34]. We exploited Mendelian rules to phase SNP genotypes in sons (GII and GIII), and linkage information to phase SNP genotypes in sires (GI and GII). CO events were then identified as phase switches in the gametes transmitted by the GII sires to their GIII sons. Double-CO occurring in intervals that were separated by less than three informative markers were attributed to genotyping errors and ignored. CO in 2-Mb windows for which the recombination rate of the GII sire was significantly >5% were attributed to GII phasing errors and ignored. The distribution of CO-events was surveyed using a graphical interface to identify as many other artifacts as possible.

### Estimating chromosome-specific proportions of meioses with 0, 1, 2, … chiasmata from the proportion of gametes with 0, 1, 2, … crossovers (CO)

Assuming absence of chromatid interference, the proportion of gametes with *i* CO from meioses with *j* chiasmata (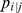), follows the binomial distribution:
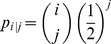
As a consequence, the proportion of gametes with *i* CO from all meiosis (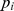) equals
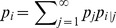
in which *p_j_* correspond to the proportion of meiosis with *j* chiasmata.

The likelihood of a dataset with *n_0_* gametes with 0 CO, *n_1_* gametes with 1 CO, *n_2_* gametes with 2 CO, etc. equals
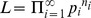
L is a function of the unknown parameters *p_j_*. We determined the values of *p_j_* that maximized L. Values considered for *j* were limited to six.

### Measuring and normalizing 60-Kb window-specific recombination rates

The recombination rate in a defined 60 Kb window was computed as 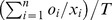 where *n* is the total number of CO events identified on the corresponding chromosome in the analyzed population, *x_i_* is the size (in bp) of the marker interval to which CO *i* has been mapped, *o_i_* the overlap (in bp) between the 60-Kb window and CO interval *i*, and *T* is the total number of analyzed gametes. To normalize window-specific recombination rates for local marker density and informativeness, we simulated (1,000 times) genotypes for the GIII sons by randomly “dropping" CO events on the phased GII chromosomes assuming a uniform distribution of CO events following a Poisson process (with mean corresponding to the real data), randomly sampling one of the two paternal chromosomes, while keeping the original maternal chromosome intact. The entire phasing and CO mapping process was then reinitiated with these *in silico* generated SNP genotypes. The corresponding simulations yielded an average recombination rate with standard deviation for each window. This allowed us to express the actual recombination rate measured for a given window in standardized deviations from the mean (across simulations).

### Quantifying crossover interference

Chromosome-specific levels of CO interference were quantified using the shape parameter 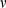 of a gamma distribution, following Broman and Weber [26]. We determined – for each chromosome – (i) the frequency distribution of CO events per gamete, (ii) the CO position (in cM) for gametes with one CO, (iii) the inter-CO distance for gametes with two CO, (iv) the inter-CO distance for gametes with three CO. We then compared these distributions with theoretical expectations under various levels of interference, accounting for chromosome size. To that end, we simulated series of “chiasmata" (CH) along four stranded bundles with gamma-distributed intervals. The shape parameter 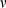 was varied from 1 (no interference) to 15 with 0.1 increments. The rate parameter was always said at 2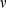. The values of the gamma variables were multiplied by 25, to obtain an average inter-CH distance of 50. The CH-series were then converted to single chromatid CO-series, by retaining CH events with a probability of 0.5. The average inter-CO distance was therefore 100 (“cM"). We then randomly sampled at least 500,000 independent segments of *n* cM from these chains, where *n* corresponds to the actual size of the studied bovine chromosome in cM. For these 500,000 “gametes", we computed the frequency distribution of (i) CO-events, (ii) CO-position for gametes with one CO (5 cM bins), (iii) inter-CO distance for gametes with two CO (5 cM bins), and (iv) inter-CO distance for gametes with three CO (5 cM bins). We then evaluated the goodness-of-fit between the real and simulated data by maximum likelihood. The likelihood of the data (*L*) was assumed to be:
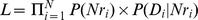
in which *N* is the total number of studied gametes, *Nr_i_* is the number of CO-events characterizing gamete *i*, *P(Nr_i_)* is the probability of having *Nr* CO-events (which is determined by the value of 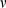), *D_i_* is the CO-position (gametes with one CO) or inter-CO distance(s)(gametes with >1 CO), and 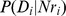 is the probability of *D_i_* given *Nr_i_* (which is determined by the value of 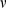). 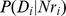 was computed for gametes with 1, 2 and 3 CO and set at 1 for the other gametes (there is no additional information to be extracted from gametes with 0 CO; gametes with >3 CO are rare and their information likely to be less reliable). For simplicity, the probability of the two inter-CO distances for gametes with two CO were considered independent.

Accordingly, this likelihood equation can be reformulated as:
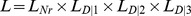
in which *L_Nr_* is the likelihood of the observed frequency distribution of number of CO per gamete, and equals 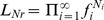, where *f_i_* is the expected frequency (given 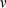) of gametes with *i* CO (given 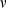) and *N_i_* is the observed number of gametes with i CO; 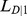 is the likelihood of the observed frequency distribution of CO-positions for gametes with one CO, and equals 
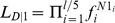
, where *f_i_* is the expected frequency (given 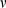) of single-CO gametes with CO-position *i* (5 cM bin), *N1_i_* is the observed number of single-CO gametes with CO-position in bin *i*, and *l* is the length (in cM) of the considered chromosome; 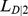 is the likelihood of the observed frequency distribution of inter-CO distance for gametes with two CO, and equals 
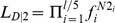
, where *f_i_* is the expected frequency (given 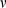) of inter-CO distance *i* (5 cM bin) for double-CO gametes, *N2_i_* is the observed number of inter-CO distance in bin *i* for double-CO gametes, and *l* is the length (in cM) of the considered chromosome; 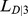 is the likelihood of the observed frequency distribution of inter-CO distance for gametes with three CO, and equals 
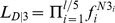
, where *f_i_* is the expected frequency (given 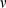) of inter-CO distance *i* (5 cM bin) for triple-CO gametes, *N3_i_* is the observed number of inter-CO distance in bin *i* for triple-CO gametes, and *l* is the length (in cM) of the considered chromosome.

The values of *f_i_* needed to compute the corresponding likelihoods were obtained from the simulations performed under varying values of 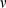.

The inter-CO distance for gametes with two CO events appeared to be the most influential parameter in determining 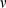 (Figure S3). We therefore focused on this measure to perform genetic analysis and QTL mapping (see also hereafter) of crossover interference. Inter-CO distances for gametes with two CO were measured in centimorgan (“GIL_cM_") or in base-pairs (“GIL_bp_"), and were normalized by subtracting the mean inter-CO distance for that chromosomes and multiplying by the standard deviation.

### Correcting GRR for family size

We noted that estimates of GRR decreased with increasing family size (Figure S4C) and attributed this to errors in determining the sire's phase. To correct GRR for this factor we used 10 paternal half-sib families with >100 G III sons. From these families we randomly sampled (1,000 times) from 1 to 10 sons with corresponding SNP genotypes. Phasing of the GI, GII and GIII bulls was conducted with Phasebook on these purposely limited data-sets, including determination of CO events in the paternal gametes transmitted to GIII sons. For each of the 10 families we then compared average GRR estimated with 1, 2, … 10 sons (over the 1,000 simulations) with GRR estimated with all sons (>100), yielding a set of 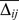 values where *i* corresponds to the number of used sons (1 to 10) for family *j*. These values were averaged across families to generate 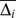, i.e. an overall effect on GRR of family size *i*, used to correct the actual GRR estimates obtained from families with <10 half-sibs.

### Estimating h^2^

Narrow sense heritabilities (*h^2^*) of recombination phenotypes (measured in the GIII sons) were estimated using two mixed models [48]. The first modeled average phenotypes of GII sires, and included an overall mean, a random individual animal effect (with variance-covariance structure proportionate to twice the coefficient of kinship between corresponding GII sires), and a random error proportionate to the inverse of the number GIII sons per GII sire. The second modeled the individual phenotypes of the gametes transmitted to GIII sons. It included an overall mean, a random individual animal effect (with variance-covariance structure proportionate to twice the coefficient of kinship between corresponding GII sires), a random permanent GII sire effect, and a random error. Variance components were estimated by restricted maximum likelihood (REML) analysis [49].

### QTL mapping

QTL were mapped using a previously described mixed model approach that simultaneously exploits linkage and LD information [34]. At each SNP position, homologues in the data set were assigned to one of 20 hidden states corresponding to “ancestral haplotype clusters". The utilized mixed models was the same as the first one used to estimate *h^2^* (i.e. modeling average phenotypes of the GII sires and adjusting the random error such that it would be proportionate to the inverse on the number of GIII observations per GII sire), with addition of a random “ancestral haplotype cluster" effect. The covariance between the effects of the 20 possible “ancestral haplotype clusters" was assumed to be zero. Significance thresholds were empirically determined by phenotype permutation [50], following standard guidelines [51]. Phenotypic values were permuted amongst half-sibs, a genome-scan conducted, and the highest (across the genome) value of the likelihood ratio test (LRT) stored. QTL were considered significant if the corresponding LRT exceeded the 95% percentile of the LRT-values obtained by permutation (i.e. if it exceeded the value of the LRT expected to occur by chance alone once every twenty genome scans). QTL were considered suggestive if the corresponding LRT exceeded the 63% percentile of the LRT-values obtained by permutation. To see the latter, a LRT that is not exceeded in 100−63 = 37% of genome scans is exceeded on average once per genome scan as 0.37 = e^−1^ (assuming that such events are Poisson distributed). A linkage signal is defined as being suggestive if it is obtained by chance alone on average once per genome scan.

### Scanning the genome for cis-acting haplotype effects on local recombination rate

To identify cis-acting haplotype effects on local recombination rate, we defined 800 Kb windows centered around the interrogated marker position. At that marker position, we selected the GII sires that were heterozygous for “ancestral haplotype clusters" [34] and tested the additive effect of “ancestral haplotype cluster" of the GII sires on the recombination phenotype of their GIII sons by ANOVA. The recombination phenotype of GIII sons was defined as the probability that a paternal CO event would have occurred in the interrogated window measured as the degree of overlap between CO encompassing marker intervals and interrogated window.

### Re-sequencing positional candidate genes and genotyping of candidate QTN

We designed primer pairs to amplify and sequence either the entire gene (*REC8*), the ORF (*RNF212*, *KCNJ2*, *KCNJ16*), or the ZF array (*PRDM9-XA* and *PRDM9-XB*) (Table S2). Animals to re-sequence were selected based on the ancestral haplotype clusters they carried at the most likely position of the corresponding QTL. Amplifications, purification of the amplicons and direct sequencing of the amplicons were carried out using standard procedures. Genotyping of candidate QTN was conducted using 5′ exonuclease (Taqman) assays for *REC8* and *RNF212* (Table S3), or by amplicon sequencing for *PRDM9-XB*.

## Supporting Information

Figure S1(A) Linear relationship between chromosome length in Mb (from UMD3.0 build) and average number of CO-events for the 29 bovine autosomes. The least square regression is characterized by a Y-intercept 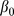 = 0.48 and a slope 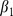 = 0.07CO/10 Mb. The slope of the regression is intermediate between the slopes characterizing male and female recombination in human [35]. (B) Proportion of meioses with zero (black), one (gray), two (blue) and three (red) chiasmata for the 29 bovine autosomes. Plain lines: proportions maximizing the likelihood of the data (assuming no chromatid interference). Dotted lines: expected proportions assuming a truncated Poisson distribution of number of chiasmata (proportion of meioses with zero chiasmata forced at zero) [36]. The data are best explained assuming near absence of nullichiasmatic meioses for autosomes 1 to 16, and frequencies <5% for the smaller chromosomes. For the largest chromosomes, the most likely (ML) frequency of meioses with at least two chiasmata is considerably higher than expected under a truncated Poisson model, supporting the preferred occurrence of a second chiasma for larger chromosomes.(PPTX)

Figure S2 (A) Representative example of the variation in male recombination in 60-Kb windows across a bovine autosome (BTA14). The plain black like (upper halve) corresponds to recombination rate estimated in the Dutch population, while the dotted black line (lower halve) corresponds to the recombination rate estimated in the NZ population. The red and blue horizontal lines correspond to “hot" and cold" windows, respectively, i.e. segments in which the observed recombination rate deviates by more than 2.5 standard deviations from the local recombination rate expected under a model of uniform distribution of CO events. (B) Variation in male recombination in 60 Kb windows across the bovine genome. The plain black like (upper halve) corresponds to recombination rate estimated in the Dutch population, while the dotted black line (lower halve) corresponds to the recombination rate estimated in the NZ population. The correlation between window-specific recombination rate in the Dutch and NZ population was high (r^2^ = 0.80; p<0.0001), despite the use of distinct SNP panels. The red and blue horizontal lines correspond to positions of “hot" and “cold" windows, respectively, i.e. segments in which the observed recombination rate deviates by more than 2.5 standard deviations from the local recombination rate expected under a model of uniform distribution of CO events. (C) Bar graphs: Frequency distribution of local (60-Kb window) recombination rate normalized for local marker density and informativeness as described in M&M. Curve: Standard normal distribution. Red and Blue vertical lines mark the thresholds defining “hot" (mean+2.5 SD) and “cold" (mean – 2.5 SD) windows, respectively. (D) Location of “hot" (red) and “cold" (blue) windows, relative to normalized chromosome length. All 29 acrocentric autosomes were aligned with their centromere towards the left of the graphs. Hot windows tend to concentrate in sub-terminal (proximal chromosome end) and terminal regions (distal chromosome end), while cold windows concentrate in the middle of the chromosome arms as well as in terminal regions (proximal chromosome end) coinciding with the centromeres.
(PPTX)

Figure S3(A) For each of the 29 bovine autosomes (BTA1-29), *column I:* frequency distribution of gametes with 0, 1, 2, … CO-events expected in the absence of cross-over interference (blue), expected given the value of ν maximizing the likelihood of the overall data (light red), expected given the value of given the value of ν maximizing the likelihood of the frequency distribution of CO-events (dark red), as observed (green). The gray bars correspond to the frequency distribution of meioses with 0, 1, 2, … chiasmata expected given the value of 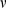 maximizing the likelihood of the frequency distribution of CO-events. The number following the BTA number corresponds to the 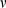-value maximizing the overall likelihood. The inset illustrates the profile of the log_10_ of the overall likelihood for varying values of 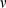. The number in brackets correspond to the 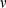-value maximizing the likelihood of the observed frequency distribution of CO number. *Column II:* Frequency distribution (5 cM bins) of position of single CO-events for gametes with one CO (green bars). The curves correspond to the distributions expected in the absence of interference (blue), assuming the 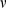-value maximizing the overall likelihood (light red), and assuming the 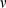-value maximizing the likelihood of the frequency distribution of single CO-positions (dark red). The numbers between brackets correspond the ν-value maximizing the likelihood of the frequency distribution of single CO-positions, and the number of observed gametes (out of a total of 7,277 used in this analysis) with one CO. *Column III:* Frequency distribution of the distance (5 cM bins) between CO events for gametes with two CO (green bars). The curves correspond to the distributions expected in the absence of interference (blue), assuming the 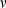-value maximizing the overall likelihood (light red), and (if different from the previous ones) assuming the 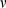-value maximizing the likelihood of the frequency distribution of inter-CO distance for gametes with two CO (dark red). The numbers between brackets correspond the 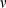-value maximizing the likelihood of the frequency distribution of inter-CO distance, and the number of observed gametes (out of a total of 7,277 used in this analysis) with two CO. *Column IV:* Frequency distribution of the distance (5 cM bins) between CO events for gametes with three CO (green bars). The curves correspond to the distributions expected in the absence of interference (blue), assuming the ν-value maximizing the overall likelihood (light red), and (if different from the previous ones) assuming the 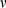-value maximizing the likelihood of the frequency distribution of inter-CO distance for gametes with three CO (dark red). The numbers between brackets correspond the 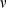-value maximizing the likelihood of the frequency distribution of inter-CO distance, and the number of observed gametes (out of a total of 7,277 used in this analysis) with three CO. (B) Chromosome-specific levels of chiasma interference measured using the shape parameter nu (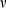) of a gamma distribution (cfr. M&M). Dark blue (All): 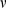-value maximizing the likelihood of all data. Red (NrCO): 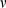-value maximizing the likelihood of the frequency distribution of CO-events per gametes. Green (SCO): 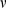-value maximizing the likelihood of the frequency distribution of CO-position (in cM) for gametes with one CO. Purple (DCO): 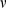-value maximizing the likelihood of the frequency distribution of inter-CO distance (in cM) for gametes with two CO. Light blue (TCO): 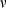-value maximizing the likelihood of the frequency distribution of inter-CO distance (in cM) for gametes with three CO. Chromosomes are ordered (left to right) from 1 to 29. The numbers under the X-axis correspond to the size of the corresponding chromosome in cM.(PDF)

Figure S4 (A) Black dots correspond to the total number of CO events identified in the paternal genome of 10,192 GIII sons sorted by GII sire. The red dots mark the average GRR for each GII sire. GRR did not differ significantly between Holstein-Friesian and Jersey bulls. (B) Correlation between the GRR estimated for 72 GII sires separately from the number of CO events transmitted to non-overlapping sets of GIII sons from H and NZ, respectively. Spearman's rank correlation was 0.58 (p<3.7×10^−7^). (C) Total number of CO events (GRR) in the genome transmitted by GII sires to their GIII sons. GIII sons are sorted according to the number of half-brothers in the data set. The increase of GRR with decreasing family size is clearly visible. (D) Lod scores obtained for GRR using 121 HF GII sires, and (i) 45 SNPs from the Illumina bovine high-density 777K SNP array mapping to the confidence interval of the BTA10 QTL (blue dots) and (ii) *REC8* SNPs (red dots). The highest lod score was obtained for *REC8* variant ss418642854. (E) Lod scores obtained for GRR using 121 HF GII sires, and (i) 27 SNPs from the Illumina bovine high-density 777K SNP array mapping to the confidence interval of the BTA6 QTL (blue dots) and (ii) *RNF212* SNPs (red dots). The highest lod score was obtained for *RNF212* variant ss469104611 ( = P259S).
(PPTX)

Figure S5 Position of the amplicons used to scan the *REC8* (A), and *RNF212* genes (B) (cfr. Table S2). The corresponding *RNF212* gene model has been submitted to Genbank.
(PPTX)

Figure S6 (A) Black dots: Average overlap (0 to 1) between marker intervals (<800-Kb) with assigned CO events and “hot" 60-K windows for GIII-sons sorted by GII-sire. Red dots: Average overlap for all CO events transmitted by corresponding GII-sire. (B) Correlation between average hot-window usage estimated for the 72 shared GII-sires respectively from gametes transmitted to Dutch versus New-Zealand GIII sons.
(PPTX)

Figure S7 (A) GIII sons inherit chromosomes with 0, 1, 2, 3, … CO from their GII sires. In this analysis, we only use “di-CO" chromosomes (i.e. with 2 CO). We measure the distance between CO-pairs in centimorgan (GIL-cM) or in base-pairs (GIL-bp) prior to normalization (i.e. expressed in standard deviations from the chromosome mean). Thus, the distance between the CO-pair of the di-CO chr. 1 inherited by son *x* from sire *y*, may be “so many" standard deviations above or below the average distance between CO-pairs on di-CO chr. 1's (across all GIII sons receiving a di-CO chr. 1 from their sire). The black dots correspond to the average of the normalized distances between CO-pairs for all di-CO chromosomes inherited by a given GIII son. GIII sons are sorted by GII sire, i.e. they are on the same vertical black line. The red dots correspond to the average of the normalized inter-CO distances across all di-CO chromosomes transmitted by a given GII sire to all its GIII sons. (B) Correlation between average normalized distance between CO events for all homologues with two recombination events transmitted by 72 shared GII-sire to their Dutch GIII-sons (X-axis), and their NZ GIII-sons (Y-axis). Inter-CO distance was measured either in centimorgan (GIL-cM) or in base pairs (GIL-bp). (C) Results of genome-scan for QTL affecting the normalized distance between pairs of CO events measured in centimorgan (GIL_cM_), using a method that simultaneously extracts linkage and LD signal^34^. The red and blue horizontal lines mark the genome-wide significant and suggestive thresholds determined by permutation testing.
(PPTX)

Table S1 (A) Following Kong et al. [8], we tested the effect of base pair composition and gene content on LRR by multiple regression. As in human, local recombination rate was positively correlated with CpG content, yet negatively correlated with GC, polyA/polyT and gene content (after adjustment for CpG content). CpG content accounted for ∼19% of the variance, while the four parameters explained ∼28% jointly.(B) We tested whether “hot" and “cold" status correlated with window content in specific interspersed repeats. For each 60-Kb hot (respectively cold) window, we sampled a “regular" window matched for CpG, GC, polyA/polyT and gene content, and compared total counts of 58 types of interspersed repeats. The statistical significance of the count difference was evaluated by permutation with Bonferroni correction for the realization of 58 independent tests. As can be seen from the table (i) some repeat types were enriched in hot and depleted in cold windows, including SINE/BovA (ratio Jungle/control: 1.12; ratio desert/control: 0.84), LTR/ERVL-MaLR (1.04;0.74), RC/Helitron (2.23;0.74) and DNA (1.30;0.75), (ii) some repeats were depleted in hot and enriched in cold windows, including LTR/ERVK (0.87;1.45), LINE/RTE-BovB (0.93;1.34) and LINE/L1 (0.98;1.06), (iii) SINE/RTE-BovB were enriched in hot and cold windows (1.12;1.06), (iv) some repeat types were depleted in hot and cold windows including SINE/tRNA-Glu (0.91;0.91), LINE/L2 (0.95;0.74), LINE/CR1 (0.92;0.64), DNA:hAT-Charlie (0.95;0.77) and DNA/MER1_type (0.95;0.67), (v) rRNA were depleted in hot windows, and (vi) SINE/MIR and satellite/centr were depleted in cold windows.
(XLS)

Table S2 Primers used for amplification and resesequencing of candidate genes *REC8*, *RNF212*, *KCNJ2*, *KCNJ16* and gonosomal *PRDM9-XA and -XB*.
(XLS)

Table S3 Primer and probes used for genotyping candidate QTN using 5′exonculease (Taqsman) assays.
(XLS)
