# Supplementary figures and images for: Genetic Variants in REC8, RNF212, and PRDM9 Influence Male Recombination in Cattle
Source: PLoS Genet. 2012 Jul 26;8(7):e1002854. doi: 10.1371/journal.pgen.1002854 (PMC3406008; doi:10.1371/journal.pgen.1002854)

## Slide 1
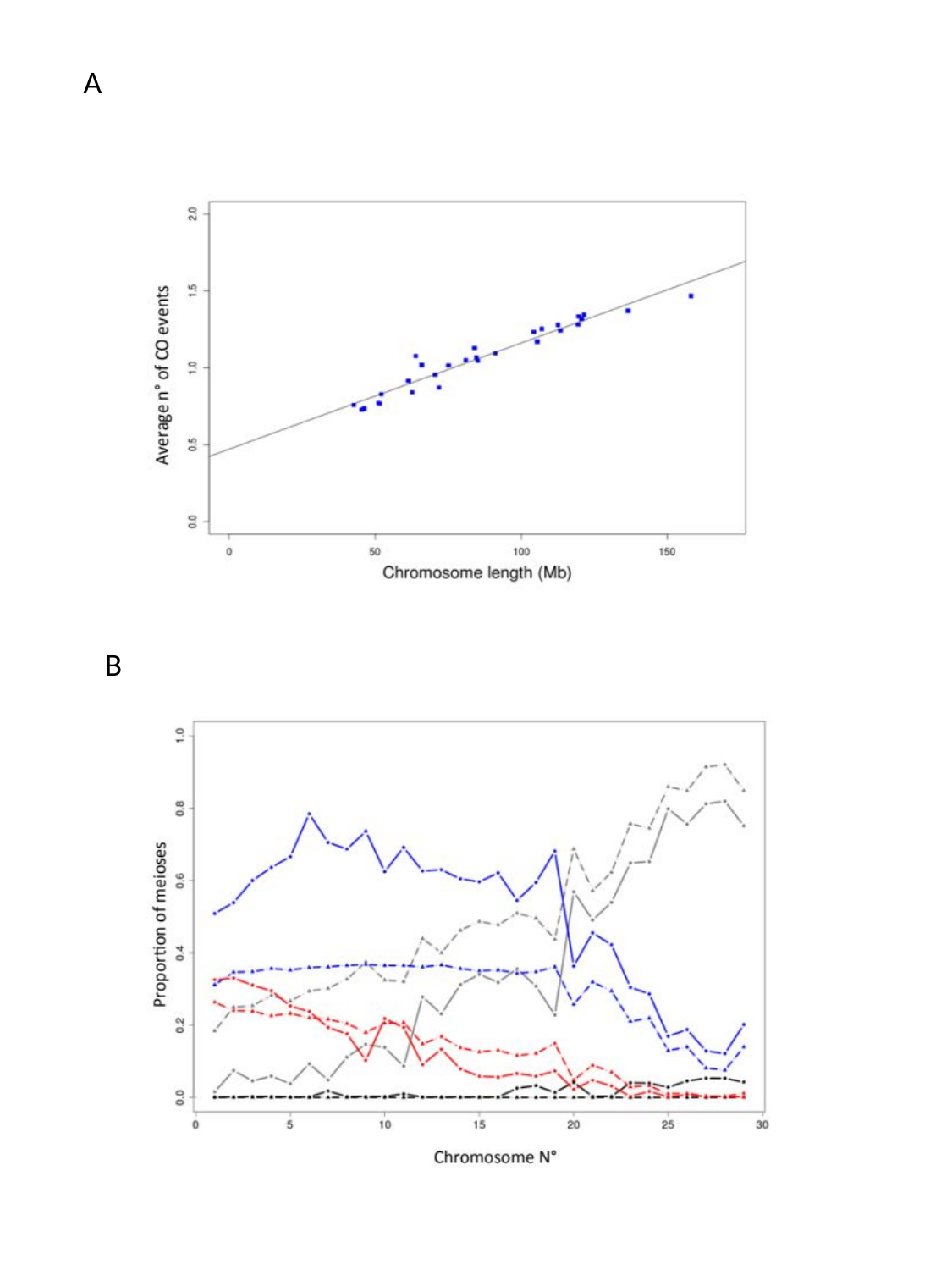

A
B

Supplement: Figure S1 — (A) Linear relationship between chromosome length in Mb (from UMD3.0 build) and average number of CO-events for the 29 bovine autosomes. The least square regression is characterized by a Y-intercept = 0.48 and a slope = 0.07CO/10 Mb. The slope of the regression is intermediate between the slopes characterizing male and female recombination in human [35]. (B) Proportion of meioses with zero (black), one (gray), two (blue) and three (red) chiasmata for the 29 bovine autosomes. Plain lines: proportions maximizing the likelihood of the data (assuming no chromatid interference). Dotted lines: expected proportions assuming a truncated Poisson distribution of number of chiasmata (proportion of meioses with zero chiasmata forced at zero) [36]. The data are best explained assuming near absence of nullichiasmatic meioses for autosomes 1 to 16, and frequencies <5% for the smaller chromosomes. For the largest chromosomes, the most likely (ML) frequency of meioses with at least two chiasmata is considerably higher than expected under a truncated Poisson model, supporting the preferred occurrence of a second chiasma for larger chromosomes. (PPTX) [file pgen.1002854.s001.pptx]

## Slide 1
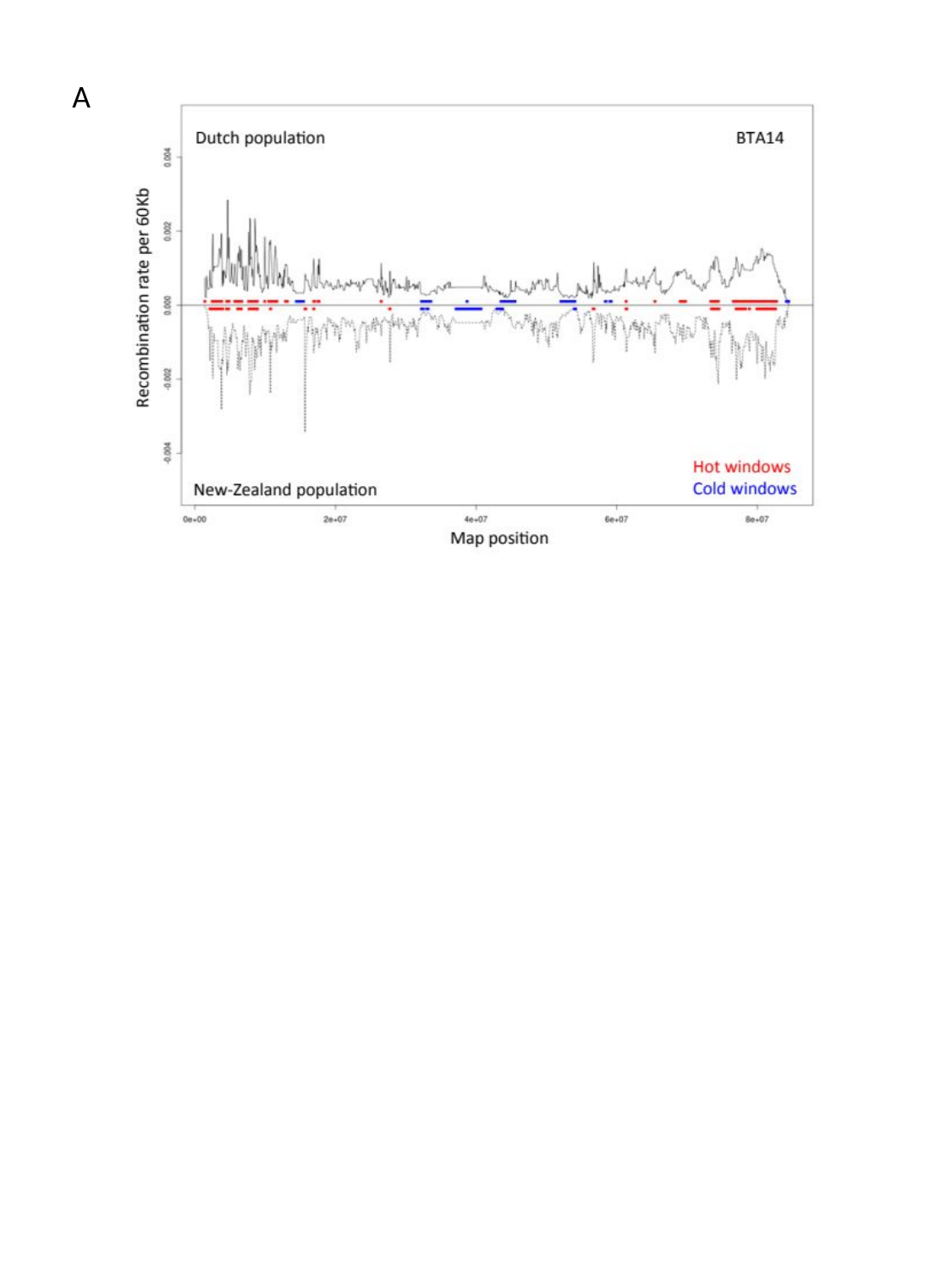

A

## Slide 2
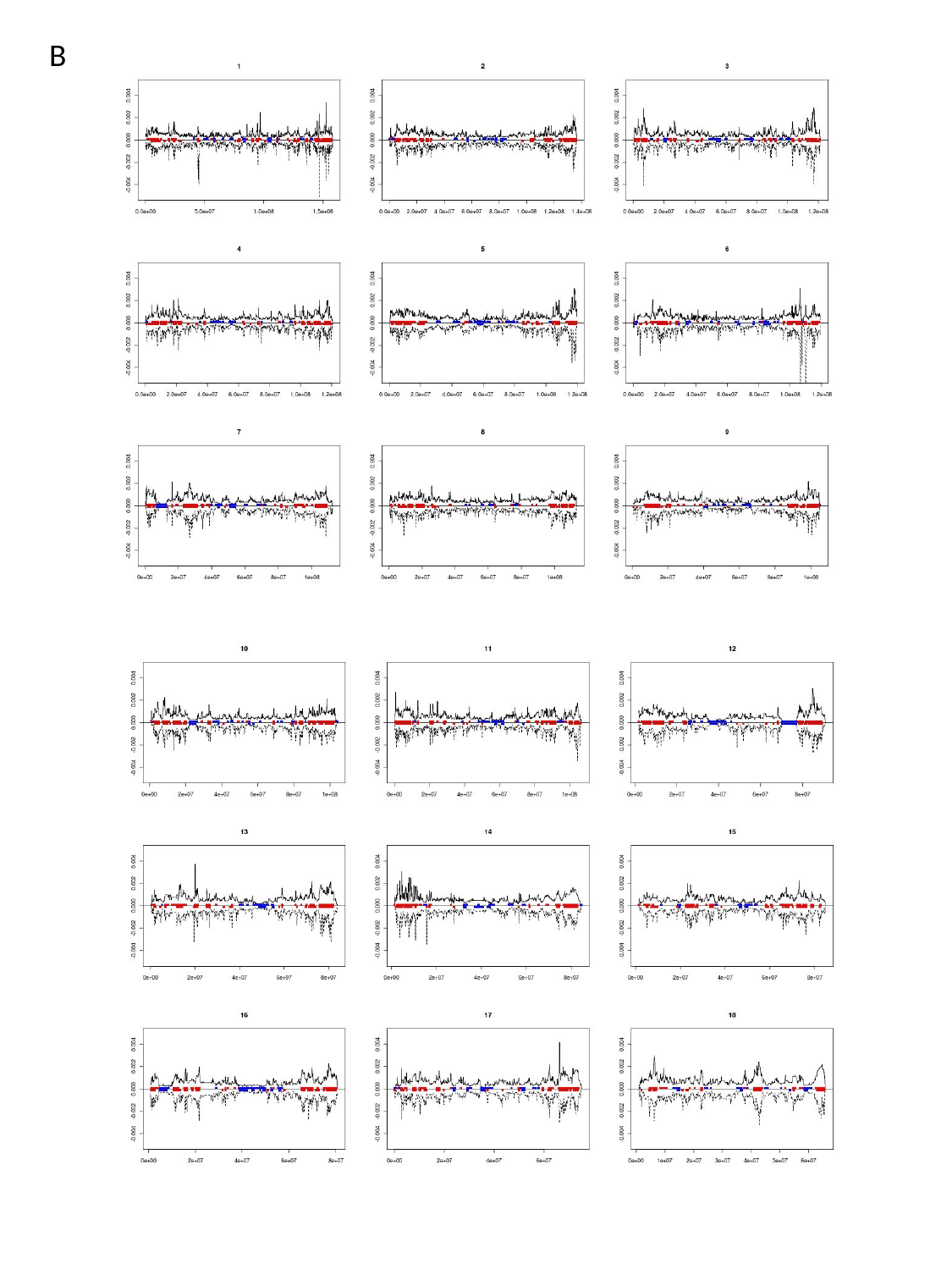

B

## Slide 3
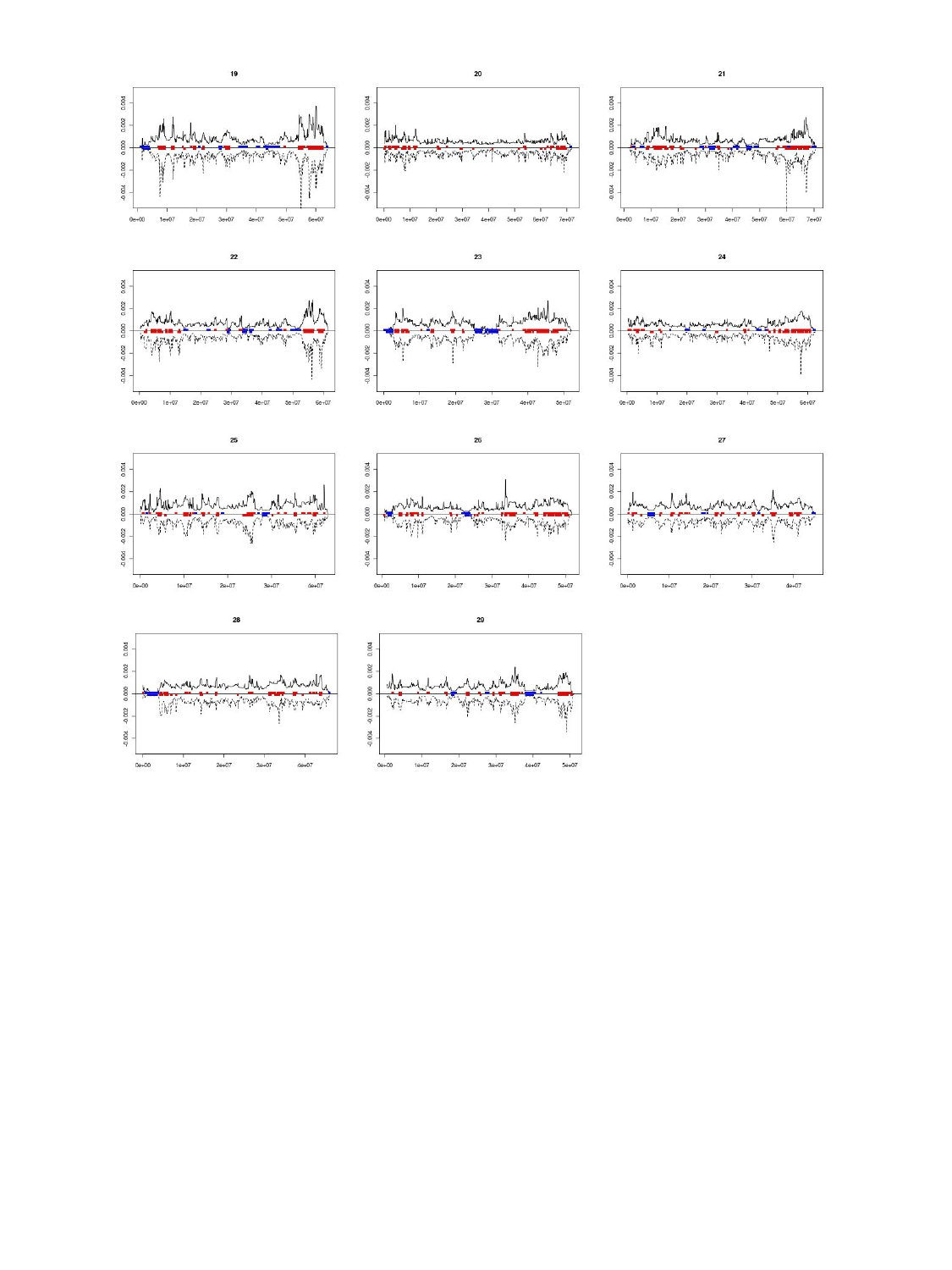

## Slide 4
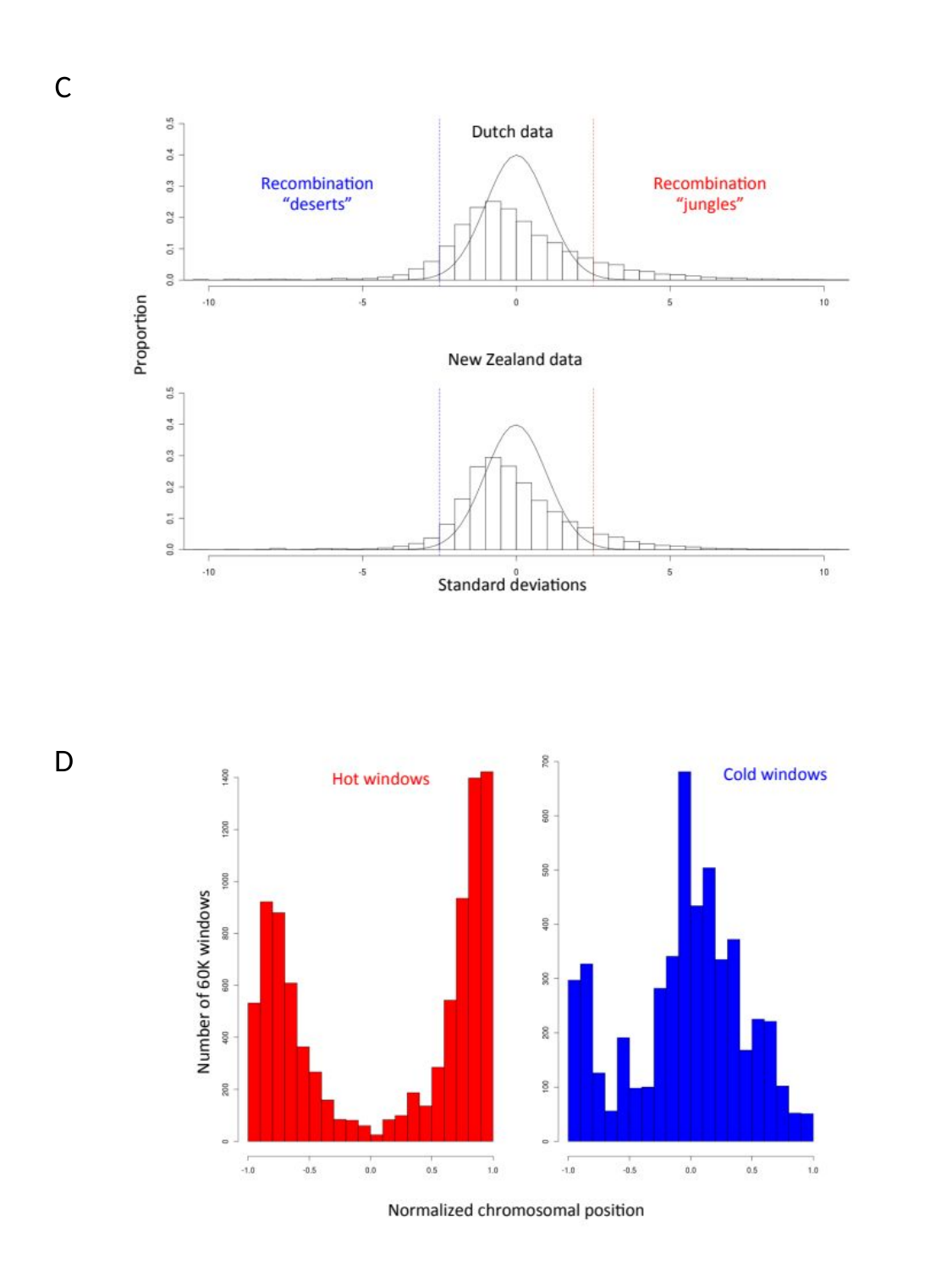

C
D

Supplement: Figure S2 — (A) Representative example of the variation in male recombination in 60-Kb windows across a bovine autosome (BTA14). The plain black like (upper halve) corresponds to recombination rate estimated in the Dutch population, while the dotted black line (lower halve) corresponds to the recombination rate estimated in the NZ population. The red and blue horizontal lines correspond to “hot" and cold" windows, respectively, i.e. segments in which the observed recombination rate deviates by more than 2.5 standard deviations from the local recombination rate expected under a model of uniform distribution of CO events. (B) Variation in male recombination in 60 Kb windows across the bovine genome. The plain black like (upper halve) corresponds to recombination rate estimated in the Dutch population, while the dotted black line (lower halve) corresponds to the recombination rate estimated in the NZ population. The correlation between window-specific recombination rate in the Dutch and NZ population was high (r2 = 0.80; p<0.0001), despite the use of distinct SNP panels. The red and blue horizontal lines correspond to positions of “hot" and “cold" windows, respectively, i.e. segments in which the observed recombination rate deviates by more than 2.5 standard deviations from the local recombination rate expected under a model of uniform distribution of CO events. (C) Bar graphs: Frequency distribution of local (60-Kb window) recombination rate normalized for local marker density and informativeness as described in M&M. Curve: Standard normal distribution. Red and Blue vertical lines mark the thresholds defining “hot" (mean+2.5 SD) and “cold" (mean – 2.5 SD) windows, respectively. (D) Location of “hot" (red) and “cold" (blue) windows, relative to normalized chromosome length. All 29 acrocentric autosomes were aligned with their centromere towards the left of the graphs. Hot windows tend to concentrate in sub-terminal (proximal chromosome end) and terminal regions (distal chromosome [file pgen.1002854.s002.pptx]

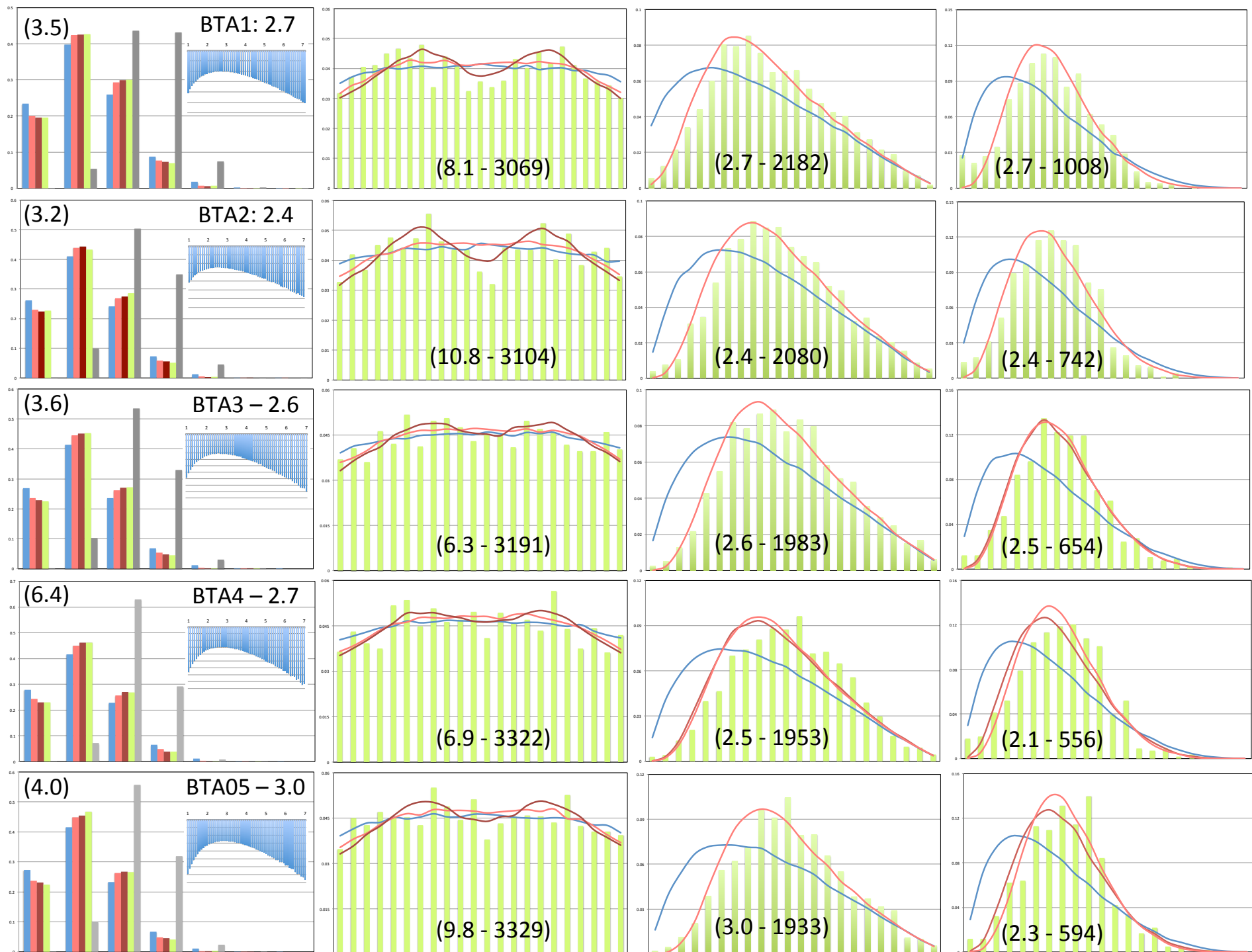

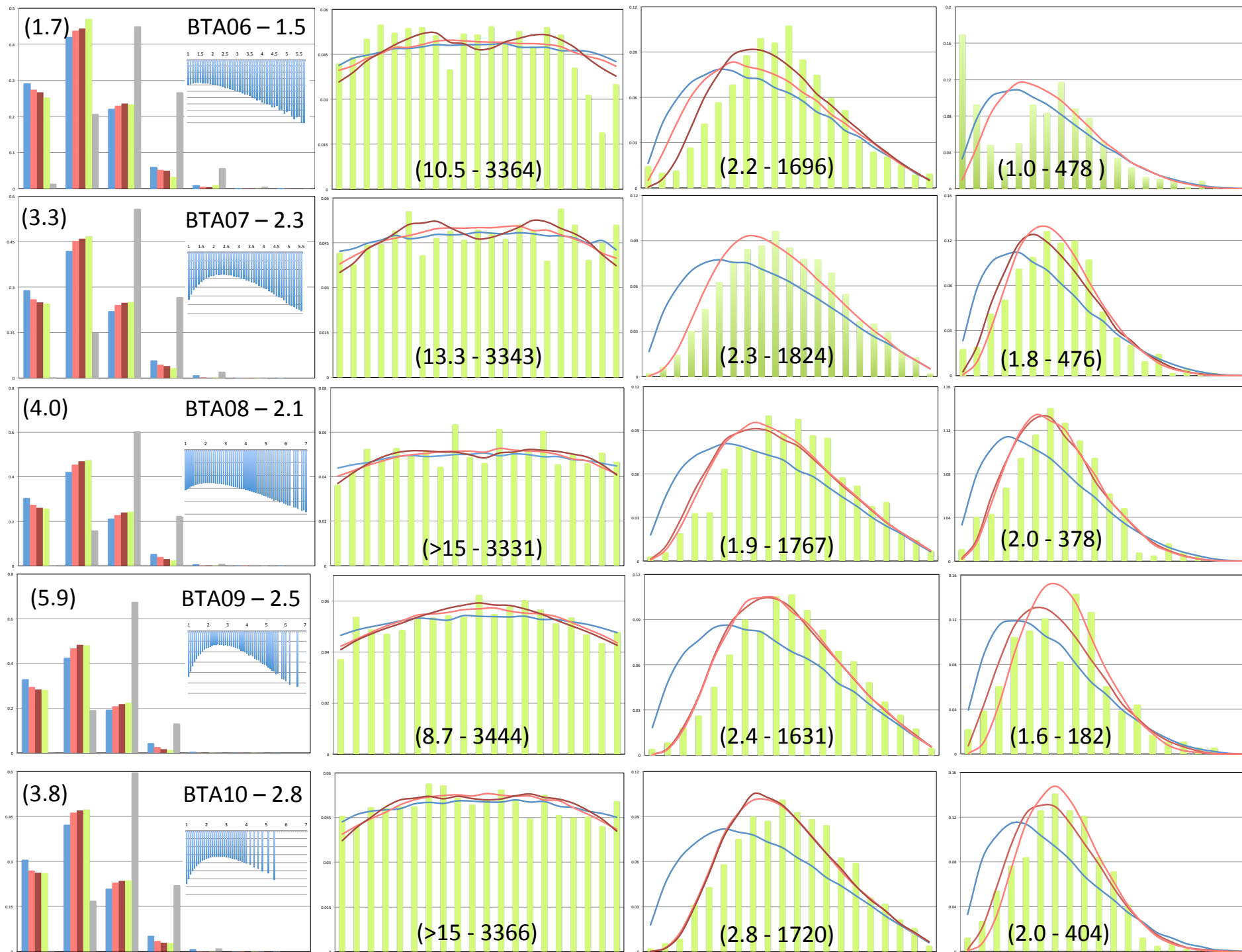

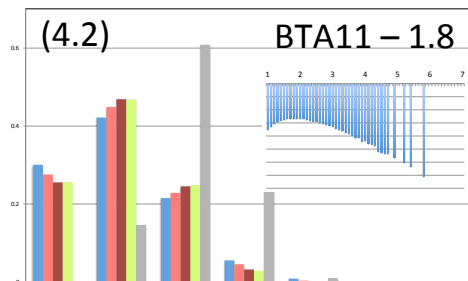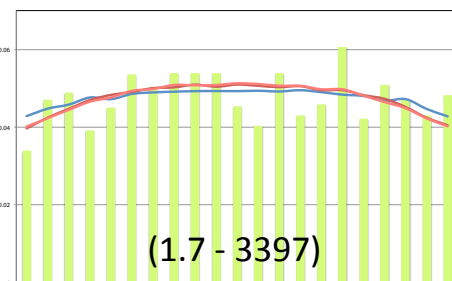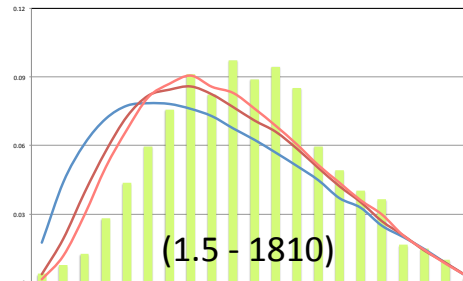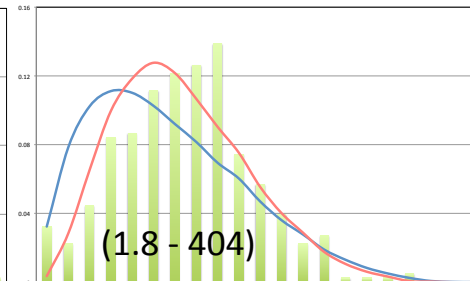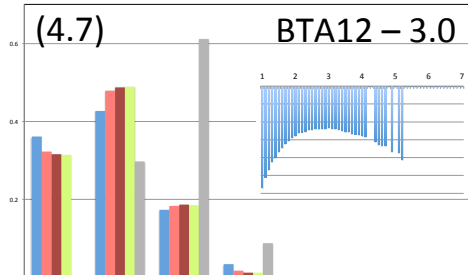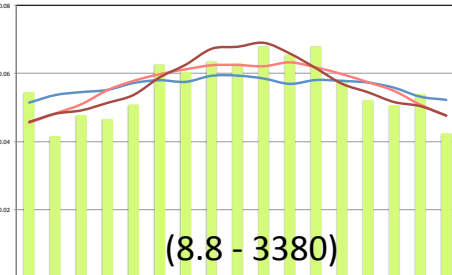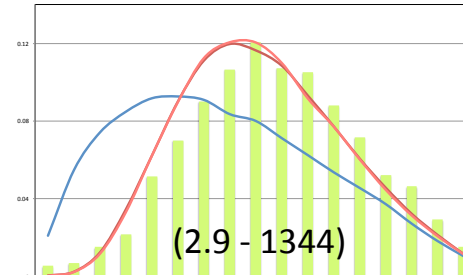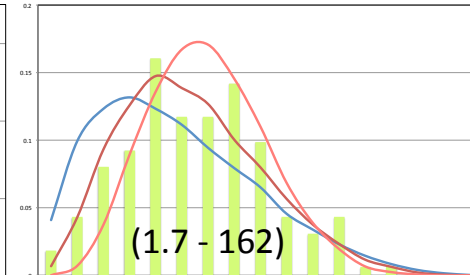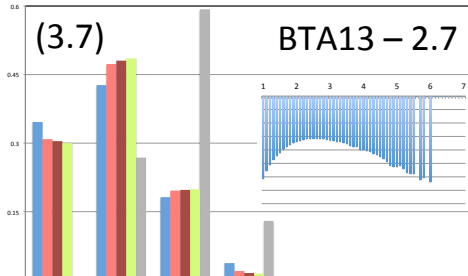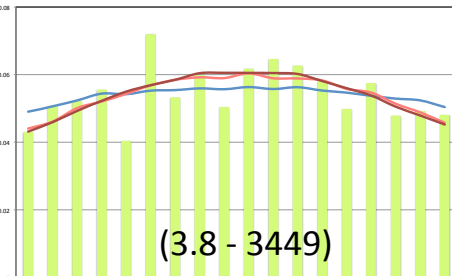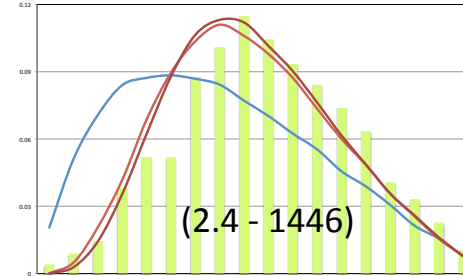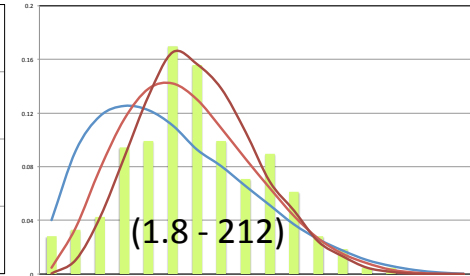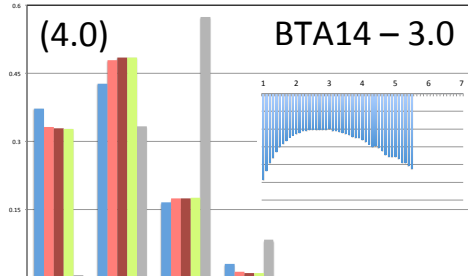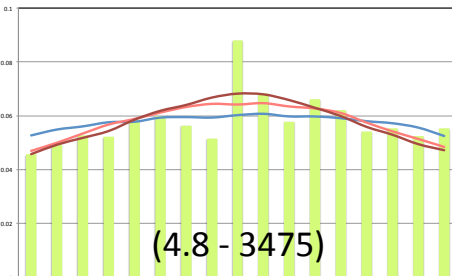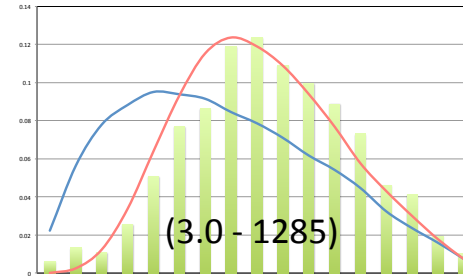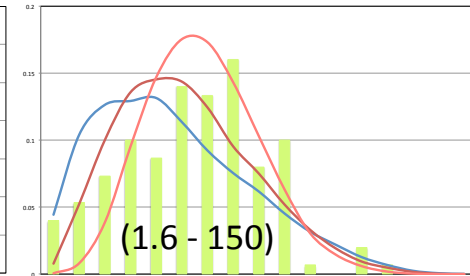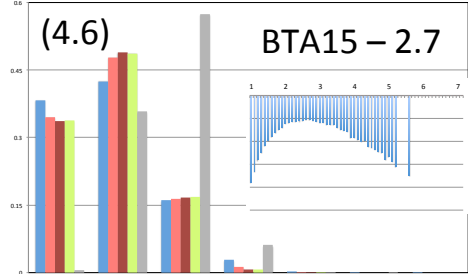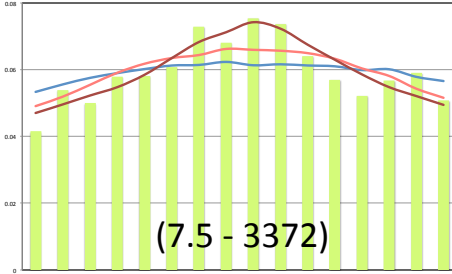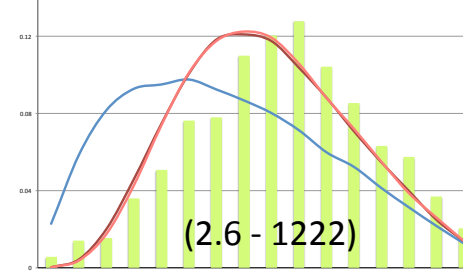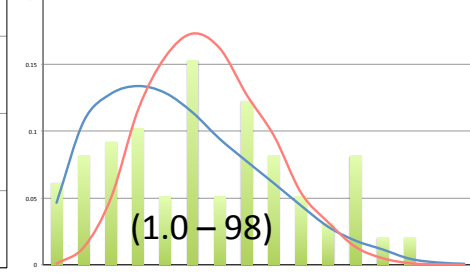

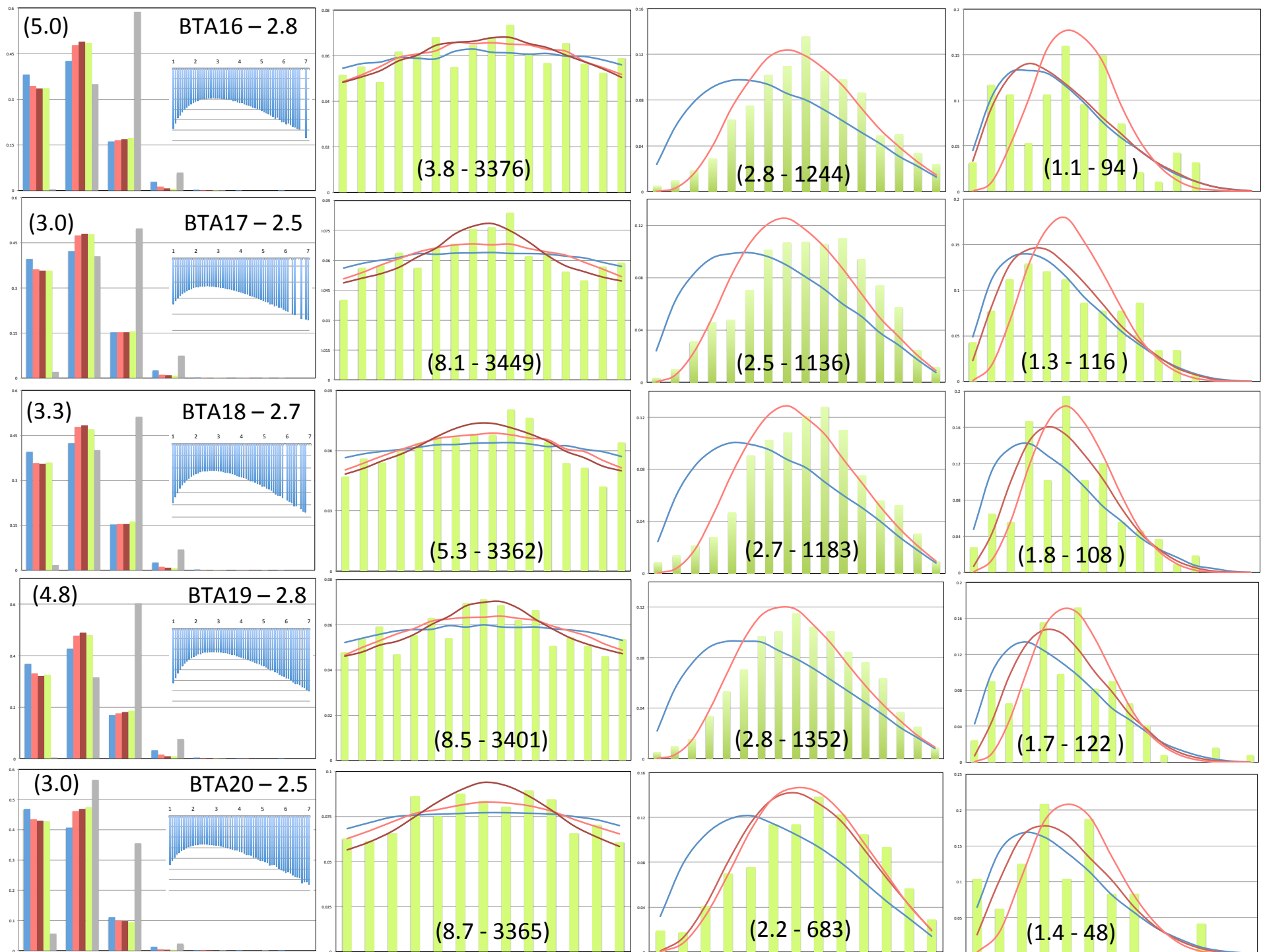

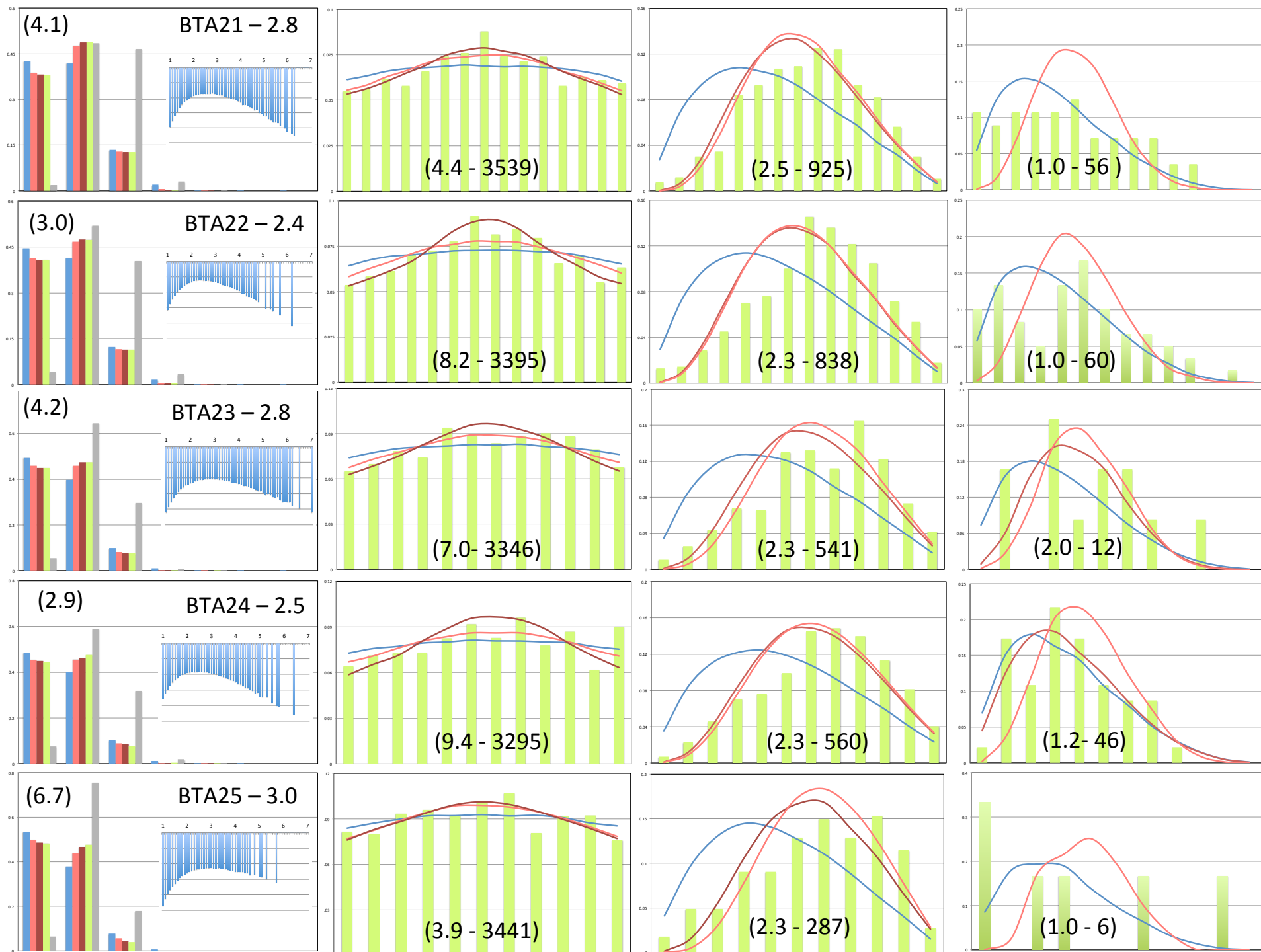

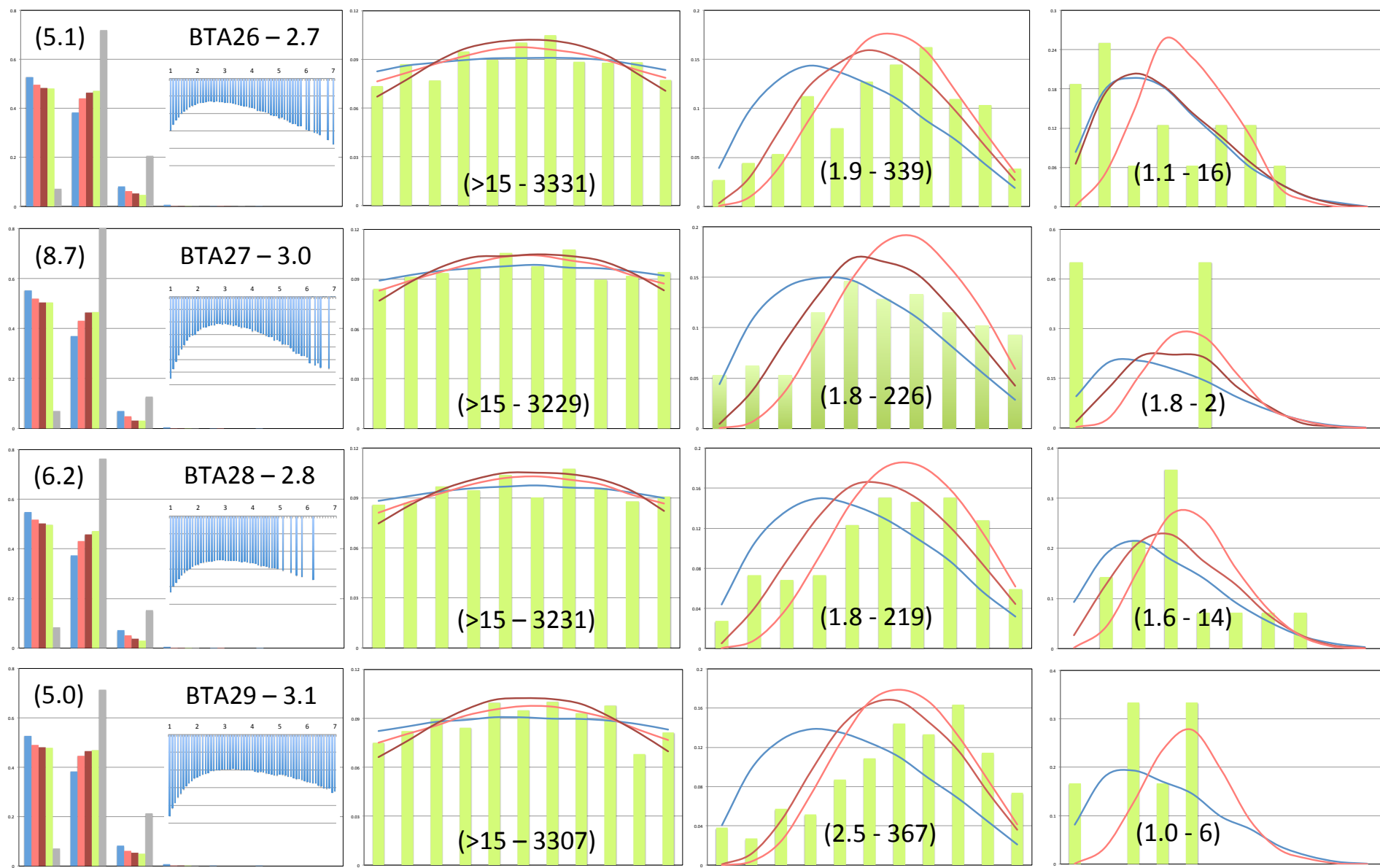

A

B

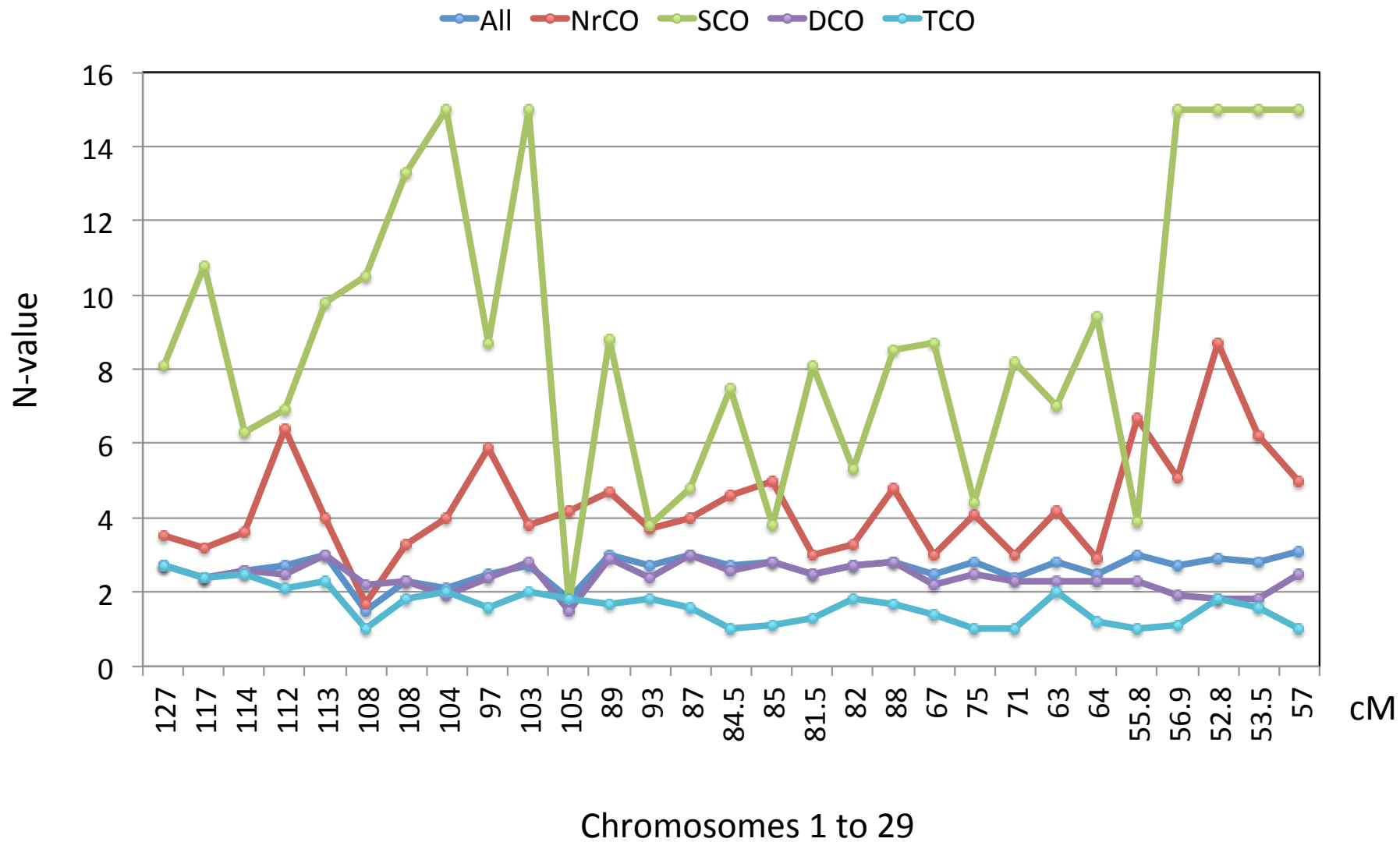

Supplement: Figure S3 — (A) For each of the 29 bovine autosomes (BTA1-29), column I: frequency distribution of gametes with 0, 1, 2, … CO-events expected in the absence of cross-over interference (blue), expected given the value of ν maximizing the likelihood of the overall data (light red), expected given the value of given the value of ν maximizing the likelihood of the frequency distribution of CO-events (dark red), as observed (green). The gray bars correspond to the frequency distribution of meioses with 0, 1, 2, … chiasmata expected given the value of maximizing the likelihood of the frequency distribution of CO-events. The number following the BTA number corresponds to the -value maximizing the overall likelihood. The inset illustrates the profile of the log10 of the overall likelihood for varying values of . The number in brackets correspond to the -value maximizing the likelihood of the observed frequency distribution of CO number. Column II: Frequency distribution (5 cM bins) of position of single CO-events for gametes with one CO (green bars). The curves correspond to the distributions expected in the absence of interference (blue), assuming the -value maximizing the overall likelihood (light red), and assuming the -value maximizing the likelihood of the frequency distribution of single CO-positions (dark red). The numbers between brackets correspond the ν-value maximizing the likelihood of the frequency distribution of single CO-positions, and the number of observed gametes (out of a total of 7,277 used in this analysis) with one CO. Column III: Frequency distribution of the distance (5 cM bins) between CO events for gametes with two CO (green bars). The curves correspond to the distributions expected in the absence of interference (blue), assuming the -value maximizing the overall likelihood (light red), and (if different from the previous ones) assuming the -value maximizing the likelihood of the frequency distribution of inter-CO distance for gametes with two CO (dark red). [file pgen.1002854.s003.pdf]

## Slide 1
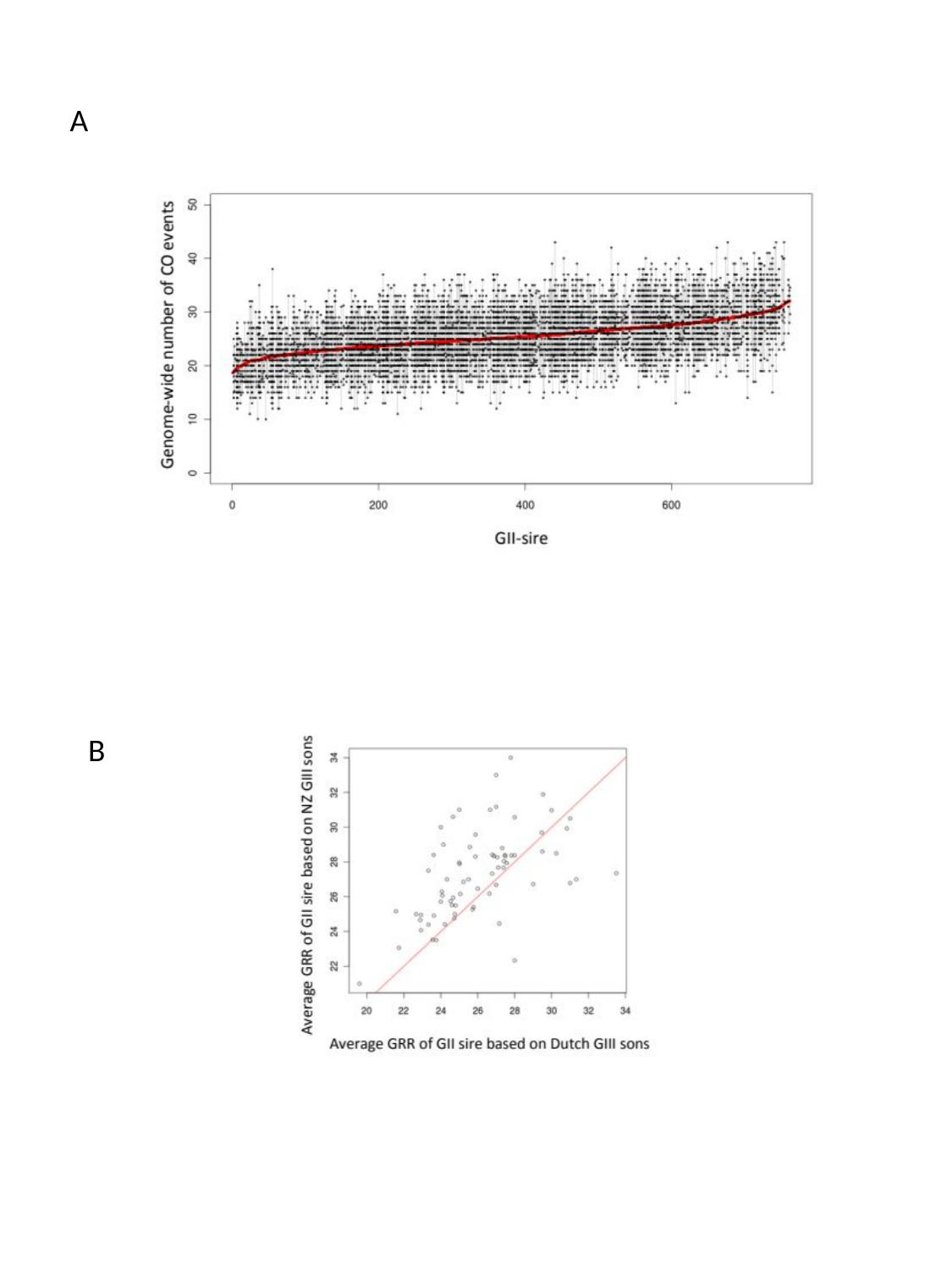

A
B

## Slide 2
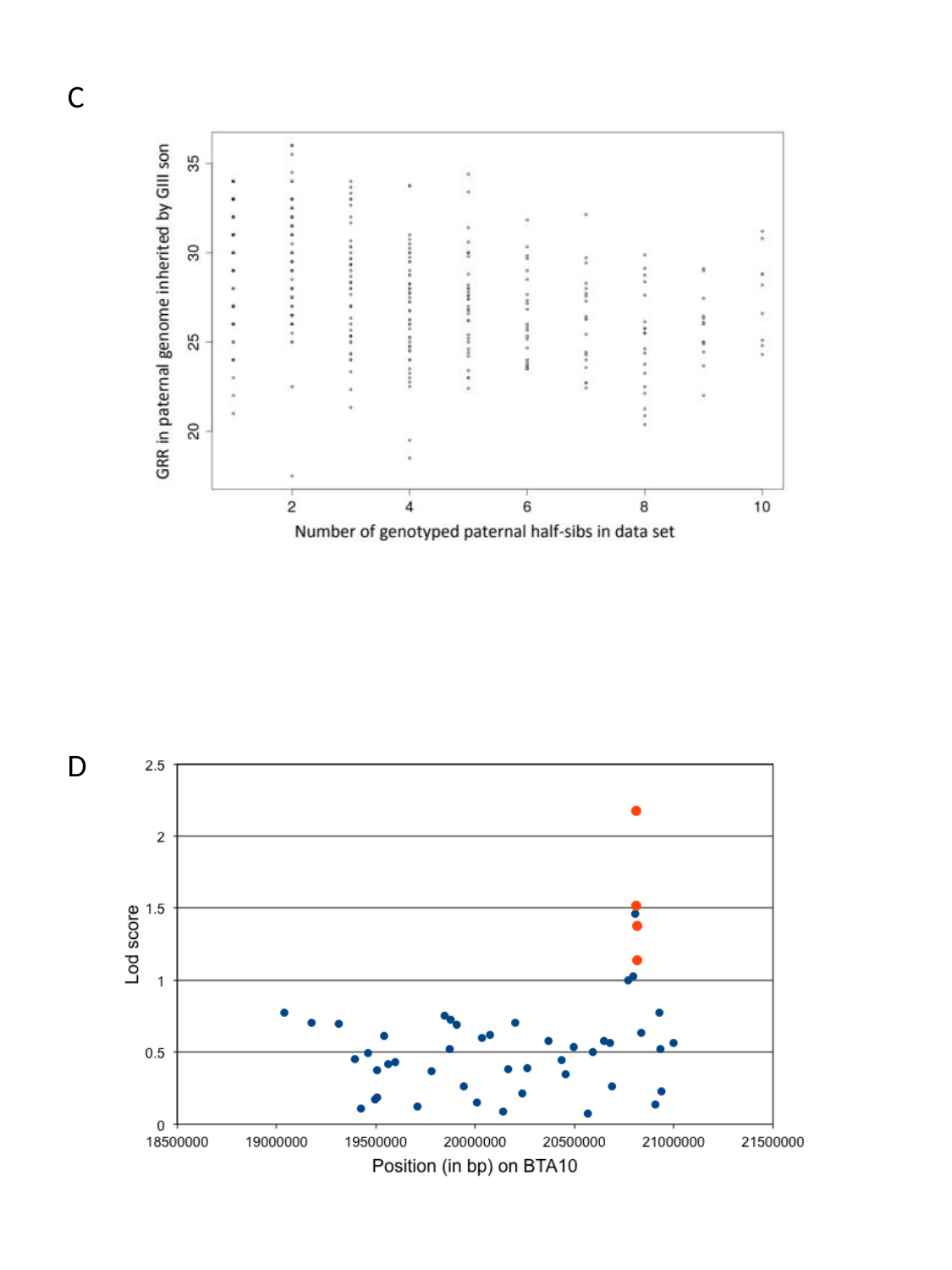

C
D

## Slide 3
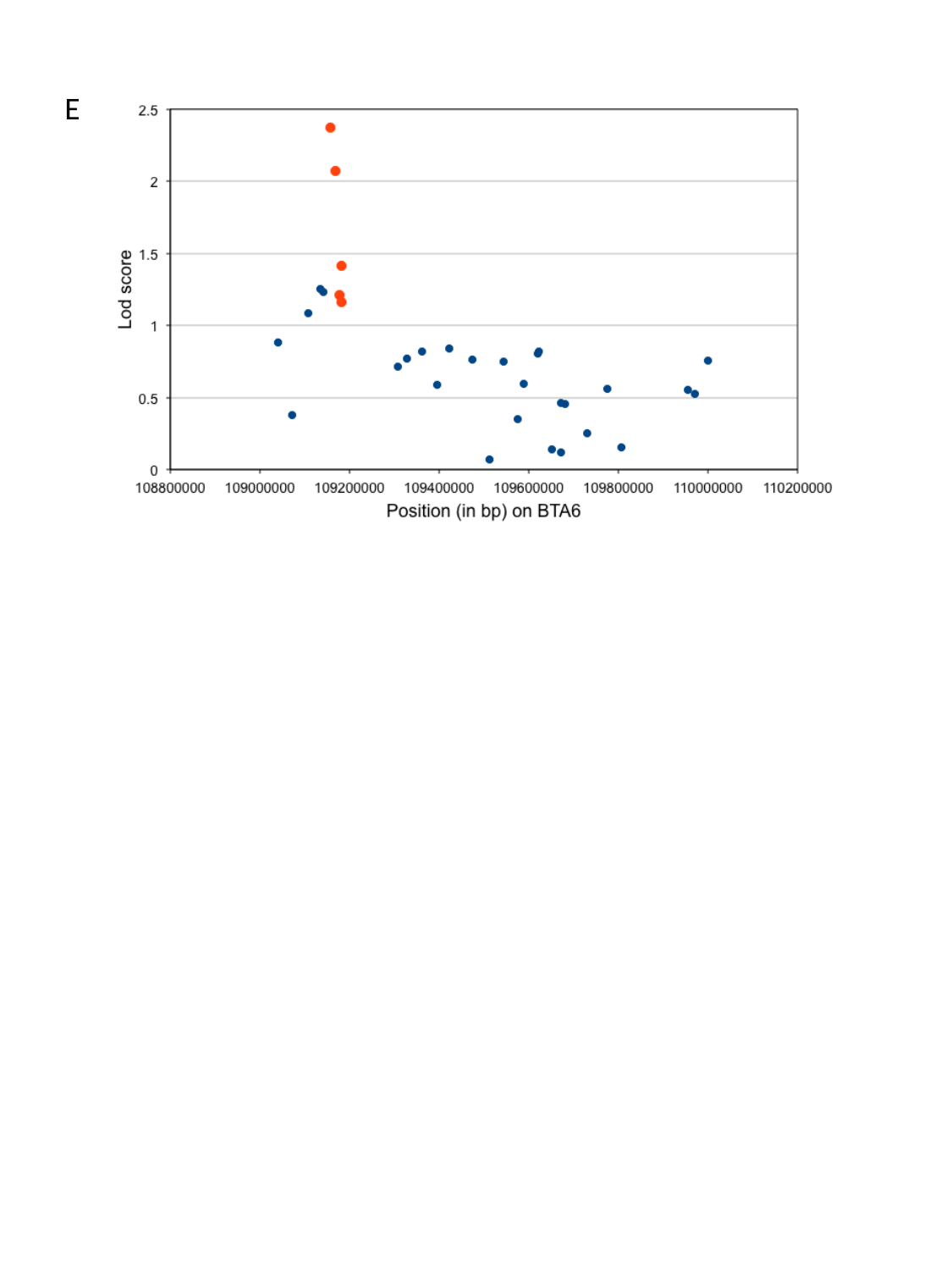

E

Supplement: Figure S4 — (A) Black dots correspond to the total number of CO events identified in the paternal genome of 10,192 GIII sons sorted by GII sire. The red dots mark the average GRR for each GII sire. GRR did not differ significantly between Holstein-Friesian and Jersey bulls. (B) Correlation between the GRR estimated for 72 GII sires separately from the number of CO events transmitted to non-overlapping sets of GIII sons from H and NZ, respectively. Spearman's rank correlation was 0.58 (p<3.7×10^−7^). (C) Total number of CO events (GRR) in the genome transmitted by GII sires to their GIII sons. GIII sons are sorted according to the number of half-brothers in the data set. The increase of GRR with decreasing family size is clearly visible. (D) Lod scores obtained for GRR using 121 HF GII sires, and (i) 45 SNPs from the Illumina bovine high-density 777K SNP array mapping to the confidence interval of the BTA10 QTL (blue dots) and (ii) REC8 SNPs (red dots). The highest lod score was obtained for REC8 variant ss418642854. (E) Lod scores obtained for GRR using 121 HF GII sires, and (i) 27 SNPs from the Illumina bovine high-density 777K SNP array mapping to the confidence interval of the BTA6 QTL (blue dots) and (ii) RNF212 SNPs (red dots). The highest lod score was obtained for RNF212 variant ss469104611 ( = P259S). (PPTX) [file pgen.1002854.s004.pptx]

## Slide 1
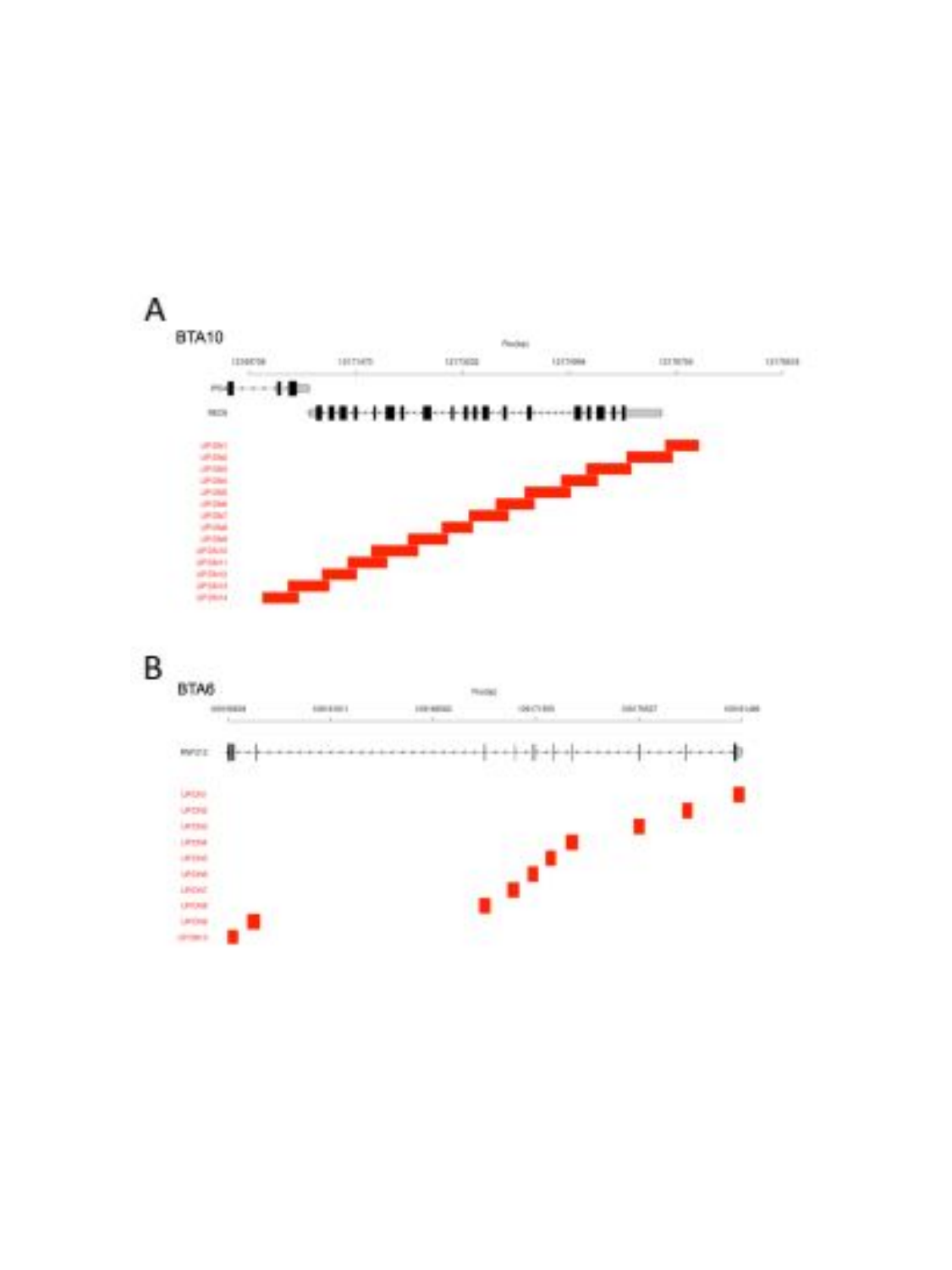

Supplement: Figure S5 — Position of the amplicons used to scan the REC8 (A), and RNF212 genes (B) (cfr. Table S2). The corresponding RNF212 gene model has been submitted to Genbank. (PPTX) [file pgen.1002854.s005.pptx]

## Slide 1
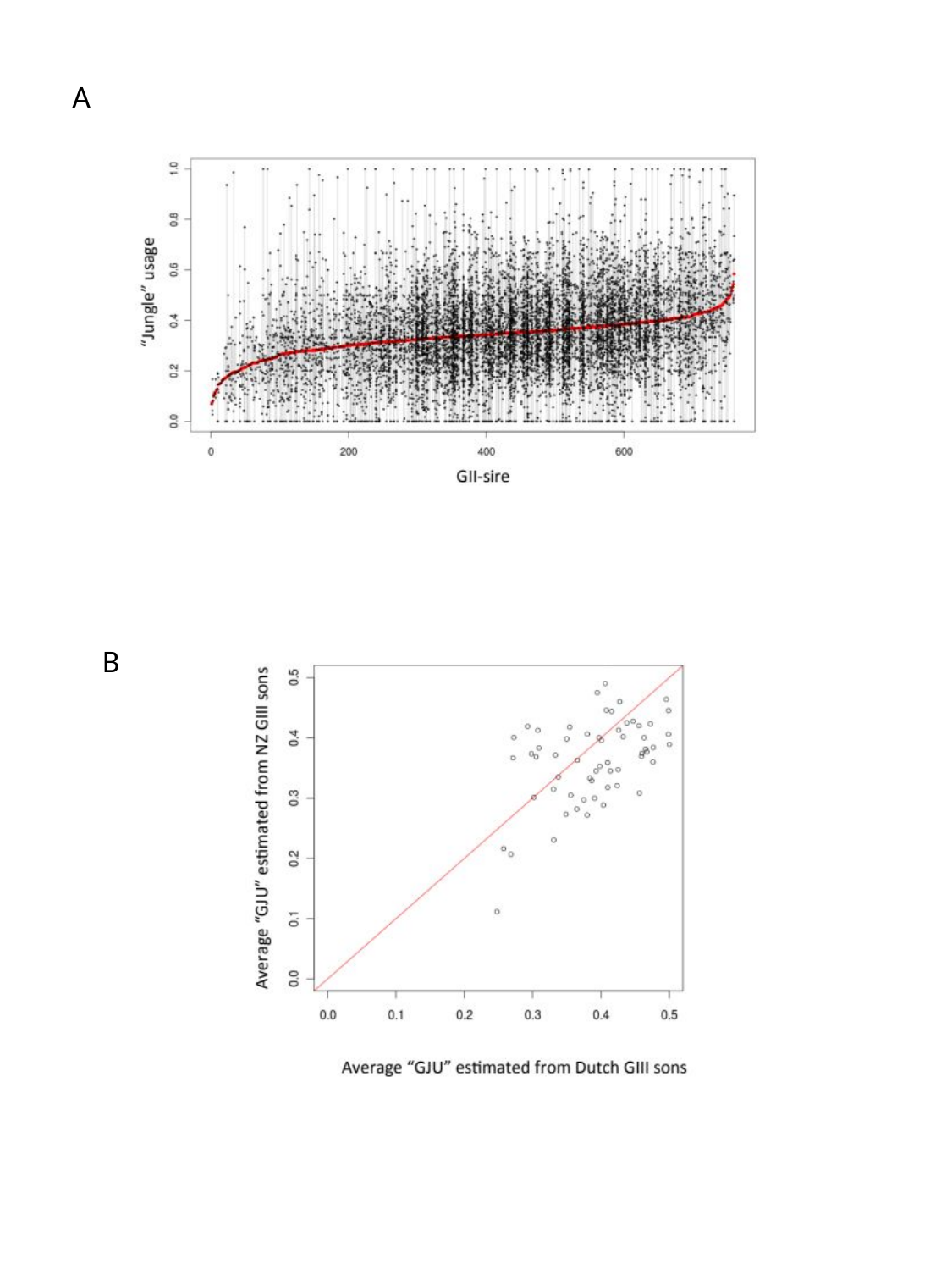

A
B

Supplement: Figure S6 — (A) Black dots: Average overlap (0 to 1) between marker intervals (<800-Kb) with assigned CO events and “hot" 60-K windows for GIII-sons sorted by GII-sire. Red dots: Average overlap for all CO events transmitted by corresponding GII-sire. (B) Correlation between average hot-window usage estimated for the 72 shared GII-sires respectively from gametes transmitted to Dutch versus New-Zealand GIII sons. (PPTX) [file pgen.1002854.s006.pptx]
