# Supplementary material for: Genetic Variants in REC8, RNF212, and PRDM9 Influence Male Recombination in Cattle
Source: PLoS Genet. 2012 Jul 26;8(7):e1002854. doi: 10.1371/journal.pgen.1002854 (PMC3406008; doi:10.1371/journal.pgen.1002854)
Supplement: Figure S7 — (A) GIII sons inherit chromosomes with 0, 1, 2, 3, … CO from their GII sires. In this analysis, we only use “di-CO" chromosomes (i.e. with 2 CO). We measure the distance between CO-pairs in centimorgan (GIL-cM) or in base-pairs (GIL-bp) prior to normalization (i.e. expressed in standard deviations from the chromosome mean). Thus, the distance between the CO-pair of the di-CO chr. 1 inherited by son x from sire y, may be “so many" standard deviations above or below the average distance between CO-pairs on di-CO chr. 1's (across all GIII sons receiving a di-CO chr. 1 from their sire). The black dots correspond to the average of the normalized distances between CO-pairs for all di-CO chromosomes inherited by a given GIII son. GIII sons are sorted by GII sire, i.e. they are on the same vertical black line. The red dots correspond to the average of the normalized inter-CO distances across all di-CO chromosomes transmitted by a given GII sire to all its GIII sons. (B) Correlation between average normalized distance between CO events for all homologues with two recombination events transmitted by 72 shared GII-sire to their Dutch GIII-sons (X-axis), and their NZ GIII-sons (Y-axis). Inter-CO distance was measured either in centimorgan (GIL-cM) or in base pairs (GIL-bp). (C) Results of genome-scan for QTL affecting the normalized distance between pairs of CO events measured in centimorgan (GILcM), using a method that simultaneously extracts linkage and LD signal34. The red and blue horizontal lines mark the genome-wide significant and suggestive thresholds determined by permutation testing. (PPTX) [file pgen.1002854.s007.pptx]

## Slide 1
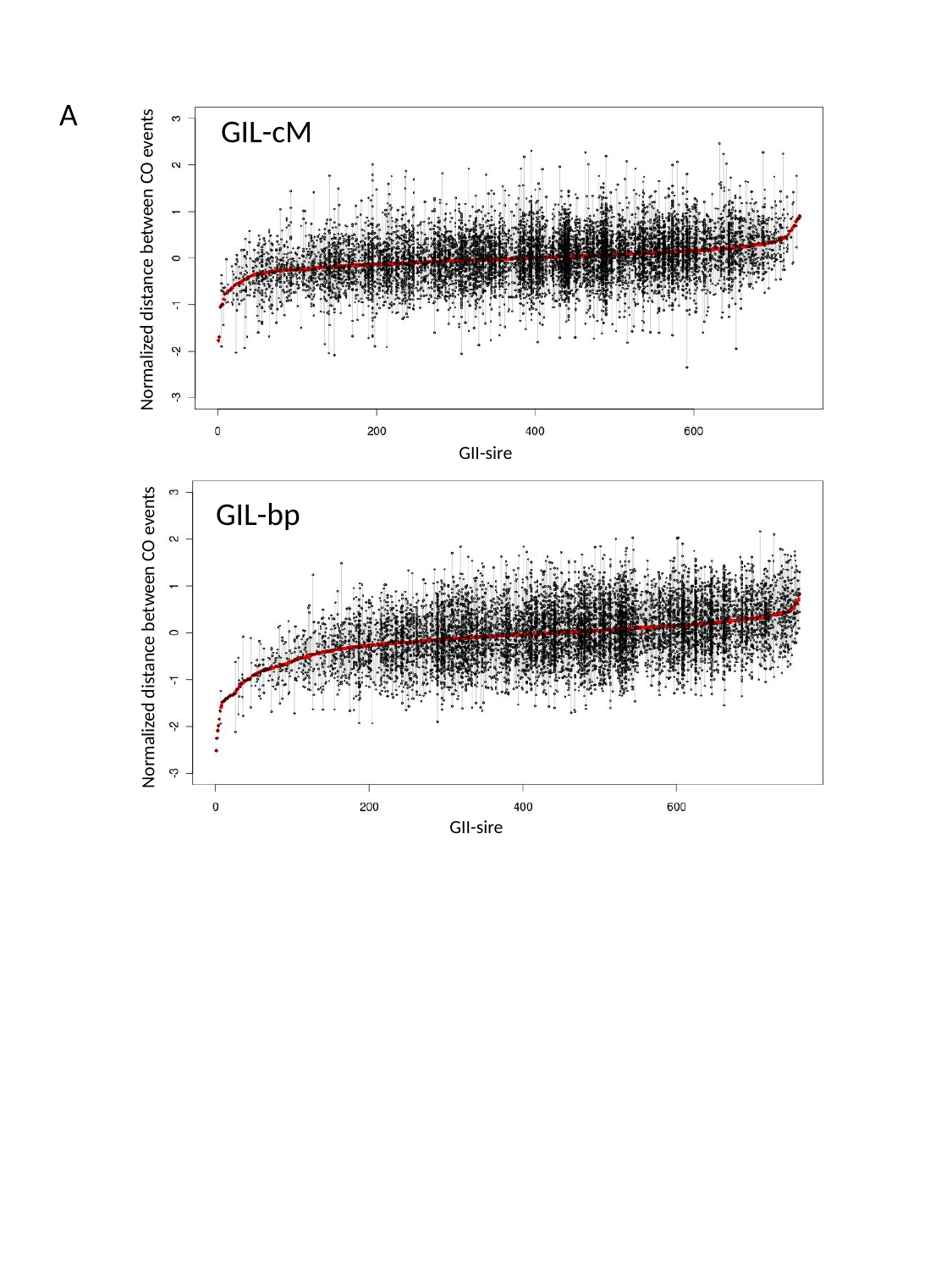

A
GIL-cM
Normalized distance between CO events
GII-sire
GIL-bp
Normalized distance between CO events
GII-sire

## Slide 2
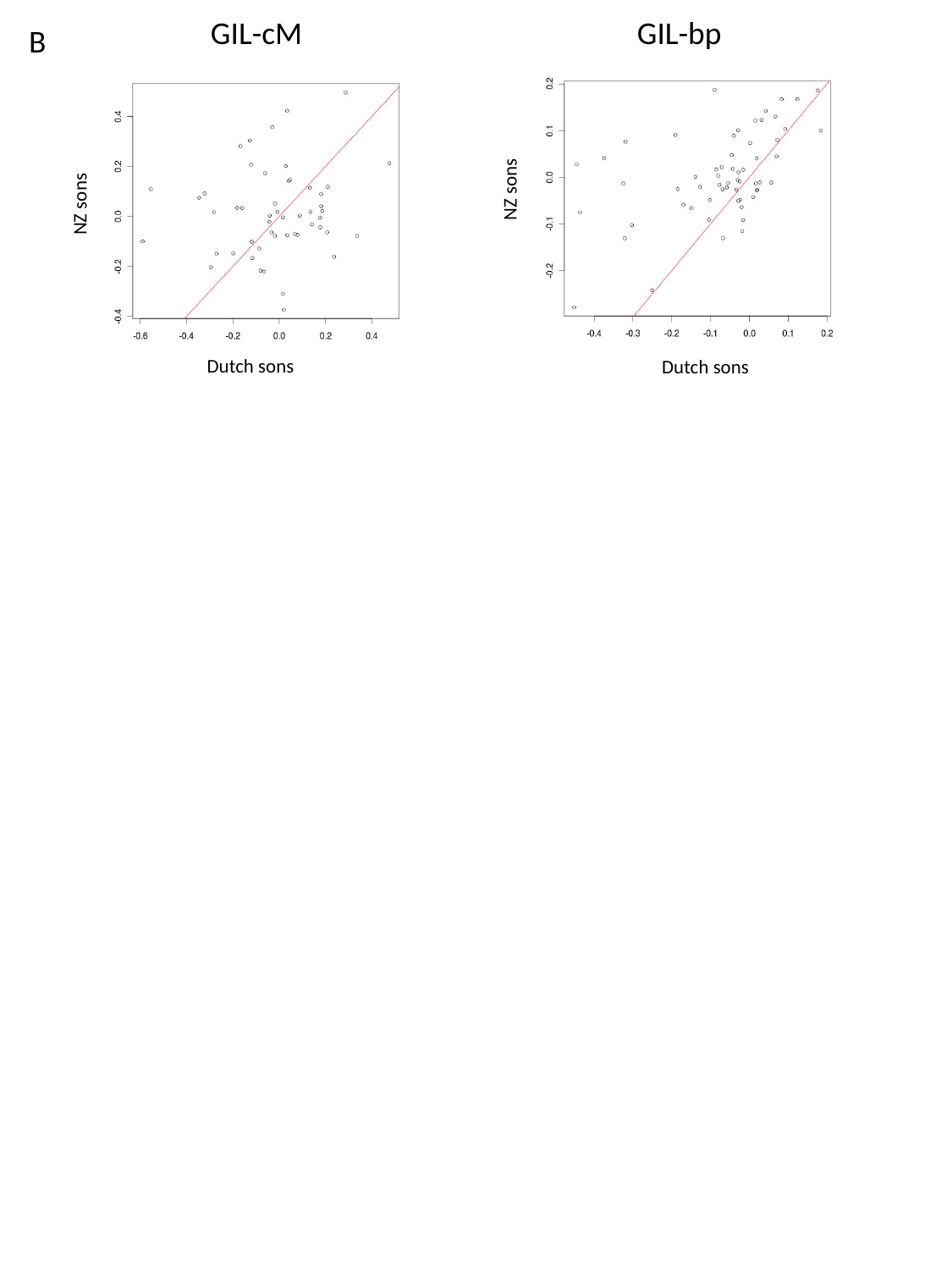

GIL-cM
GIL-bp
B
NZ sons
NZ sons
Dutch sons
Dutch sons

## Slide 3
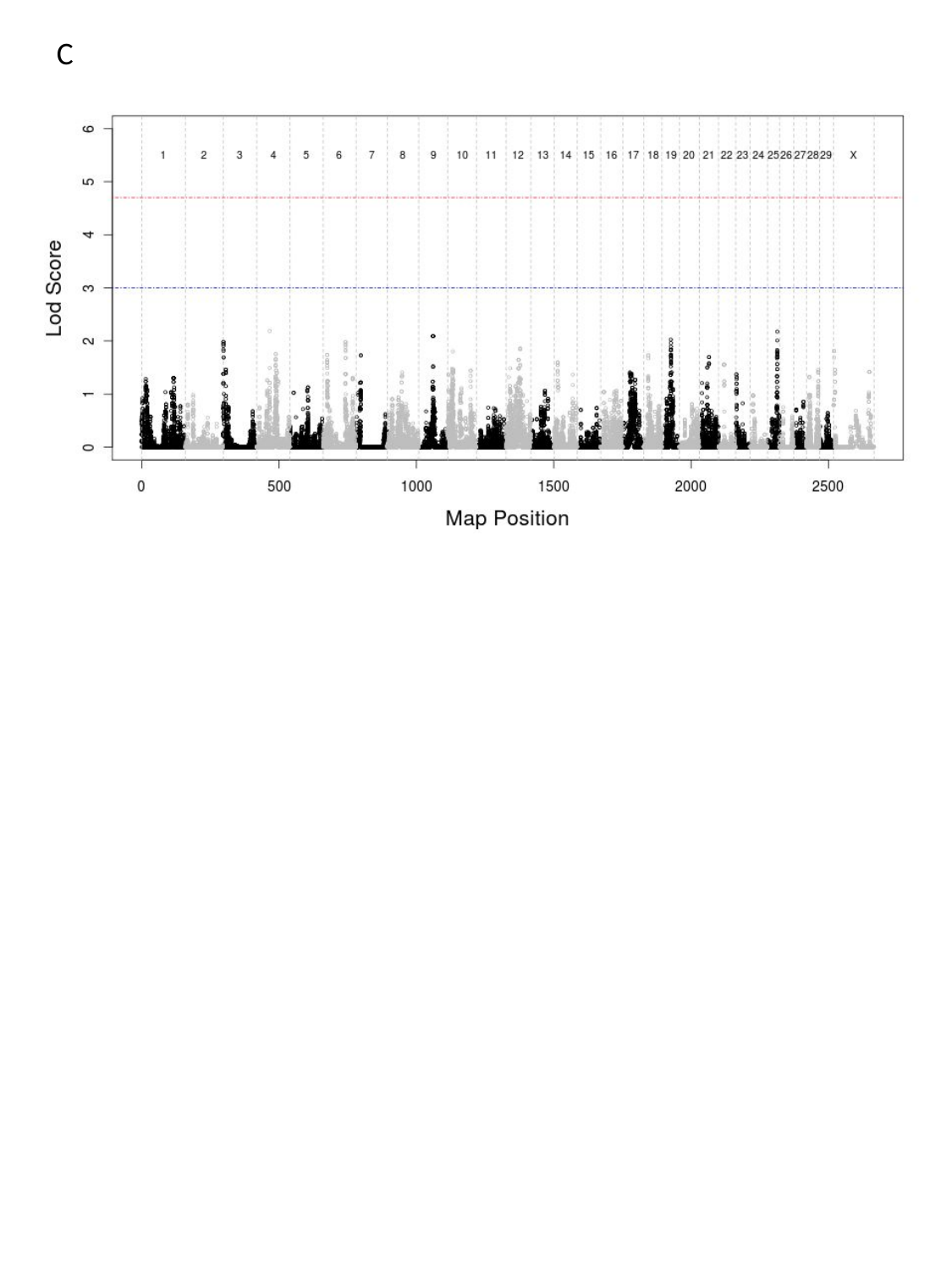

C
